# Effects of *N*-Methyl-d-Aspartate Receptor Antagonists on Gamma-Band Activity During Auditory Stimulation Compared With Electro/Magneto-encephalographic Data in Schizophrenia and Early-Stage Psychosis: A Systematic Review and Perspective

**DOI:** 10.1093/schbul/sbae090

**Published:** 2024-06-27

**Authors:** Bianca Bianciardi, Helena Mastek, Michelle Franka, Peter J Uhlhaas

**Affiliations:** Institute of Neuroscience and Psychology, University of Glasgow, Glasgow, UK; Department of Child and Adolescent Psychiatry, Charité – Universitätsmedizin Berlin, corporate member of Freie Universität Berlin and Humboldt-Universität zu Berlin, Berlin, Germany; Department of Child and Adolescent Psychiatry, Charité – Universitätsmedizin Berlin, corporate member of Freie Universität Berlin and Humboldt-Universität zu Berlin, Berlin, Germany; Institute of Neuroscience and Psychology, University of Glasgow, Glasgow, UK; Department of Child and Adolescent Psychiatry, Charité – Universitätsmedizin Berlin, corporate member of Freie Universität Berlin and Humboldt-Universität zu Berlin, Berlin, Germany

**Keywords:** gamma-band oscillations, auditory perception, EEG/MEG, schizophrenia, preclinical research, NMDA-R antagonists, ketamine, MK-801, PCP, E/I imbalance

## Abstract

**Background and Hypothesis:**

*N*-Methyl-d-aspartate receptor (NMDA-R) hypofunctioning has been hypothesized to be involved in circuit dysfunctions in schizophrenia (ScZ). Yet, it remains to be determined whether the physiological changes observed following NMDA-R antagonist administration are consistent with auditory gamma-band activity in ScZ which is dependent on NMDA-R activity.

**Study Design:**

This systematic review investigated the effects of NMDA-R antagonists on auditory gamma-band activity in preclinical (*n* = 15) and human (*n* = 3) studies and compared these data to electro/magneto-encephalographic measurements in ScZ patients (*n* = 37) and 9 studies in early-stage psychosis. The following gamma-band parameters were examined: (1) evoked spectral power, (2) intertrial phase coherence (ITPC), (3) induced spectral power, and (4) baseline power.

**Study Results:**

Animal and human pharmacological data reported a reduction, especially for evoked gamma-band power and ITPC, as well as an increase and biphasic effects of gamma-band activity following NMDA-R antagonist administration. In addition, NMDA-R antagonists increased baseline gamma-band activity in preclinical studies. Reductions in ITPC and evoked gamma-band power were broadly compatible with findings observed in ScZ and early-stage psychosis patients where the majority of studies observed decreased gamma-band spectral power and ITPC. In regard to baseline gamma-band power, there were inconsistent findings. Finally, a publication bias was observed in studies investigating auditory gamma-band activity in ScZ patients.

**Conclusions:**

Our systematic review indicates that NMDA-R antagonists may partially recreate reductions in gamma-band spectral power and ITPC during auditory stimulation in ScZ. These findings are discussed in the context of current theories involving alteration in E/I balance and the role of NMDA hypofunction in the pathophysiology of ScZ.

## Introduction

The pathophysiological processes underlying schizophrenia (ScZ), a severe psychiatric syndrome, remain to be determined. Dopaminergic neurotransmission has been implicated in the generation of positive symptoms, such as delusions and hallucinations, and is the main target for antipsychotic medications (APMs).^1^ However, APMs have only limited efficacy for negative symptoms and cognitive impairments.^2,3^ More recently, a glutamatergic dysfunction has gained attention as a plausible mechanism, which could potentially account for the developmental profile and cognitive dysfunctions associated with the disorder.^4–7^

In particular, aberrant *N*-methyl-d-aspartate receptor (NMDA-R) functioning has been implicated in circuit dysfunctions in ScZ.^4^ Expression of the NR1 subunit in the prefrontal cortex (PFC) is decreased in postmortem studies,^8^ but also other NMDA-R subunits.^9,10^ Importantly, there are findings to suggest NMDA-Rs may be reduced on gamma-aminobutyric acid (GABA) interneurons.^11^ Finally, genetic evidence has also shown that Copy Number Variants in ScZ patients are enriched for NMDA-Rs.^12^

Further evidence for the role of NMDA-Rs in the pathophysiology of ScZ comes from studies that investigated the effects of NMDA-Rs antagonists, such as ketamine and phencyclidine (PCP), in healthy volunteers.^13,14^ NMDA-R antagonists elicit transient positive, negative, and cognitive symptoms in healthy volunteers.^14,15^ In addition, acute and chronic administration of NMDA-R antagonists in animal models recreate several core circuit dysfunctions observed in ScZ,^16,17^ such as deficits in parvalbumin-expressing GABAergic interneurons.^18^

GABAergic interneurons are also critically involved in the generation of neural oscillations as the precise inhibition of pyramidal cell activity regulates the output of neuronal assemblies, leading to rhythmic fluctuations in neural excitability.^19–21^ Specifically, parvalbumin-expressing (PV+) GABAergic interneurons have been involved in the generation of high-frequency oscillations^19^ as well α-amino-3-hydroxy-5-methyl-4-isoxazolepropionic acid (AMPA)- and NMDA-R-mediated activation of PV+ interneurons.^22^

In ScZ, there is consistent evidence for alterations in both resting-state^23–25^ and task-related gamma-band (30–120 Hz) activity.^26–28^ These data in turn have been linked to changes in excitation and inhibition balance (E/I-balance) parameters in ScZ, such as the reduced levels of PV+ interneuron activity.^29,30^ Moreover, NMDA-R hypofunctioning has also been proposed to be compatible with alterations in both resting-state^31^ and task-related gamma-band parameters in ScZ.^32^

In the current study, we carried out a systematic review to test the hypothesis that NMDA-R antagonists, such as ketamine, MK-801, and PCP, in animal models and human participants recreate the pattern of changes observed in electro/magneto-encephalographic (EEG/MEG) data during auditory stimulation in ScZ. A large body of work has investigated gamma-band activity during auditory tasks, such as oddball paradigms^33,34^ as well as auditory steady-state responses (ASSR).^35,36^ There is evidence for reductions in gamma-band power in chronic ScZ patients,^37^ first-episode psychosis (FEP) and in high risk for psychosis (CHR-P) participants^38^ as well as in first-degree relatives.^39^ However, not all studies have confirmed these findings.^40–42^

To link these data with the preclinical and pharmacological literature, we systematically examined studies that investigated the effects of NMDA-R antagonists on gamma-band activity during auditory stimulation in rodent and human electrophysiological recordings with the aim to compare these changes with EEG/MEG data in ScZ and early-stage psychosis patients. Moreover, we examined the potential impact of alterations in baseline gamma-band power and its relationship to NMDA-R antagonists. Accordingly, we hypothesized that auditory gamma-band activity during NMDA-R hypofunctioning in preclinical and human EEG/MEG recordings would be compatible with the findings observed in ScZ patients as well as during early-stage psychosis.^43–45^

## Method

Publication searches were conducted in Google Scholar and PubMed using a combination of the following search terms for the preclinical literature: “Rodents”/“Monkey,” “Ketamine”/“PCP”/“MK801”/“NMDA-R,” “Gamma oscillations,” “Auditory Task” until January 2022. For human studies investigating the effects of ketamine on gamma-band oscillations, “Human,” “Ketamine,” “NMDA-R,” “Gamma,” and “Auditory Task” were entered. Additionally, studies that examined gamma-band oscillations EEG/MEG data in healthy humans as well as EEG, local field potentials (LFPs), and electrocorticography (ECoG) for preclinical studies were identified with the search terms: “EEG/MEG” and “EEG/LFP/ECoG.” Furthermore, reference lists of appropriate publications were searched to identify studies matching our search criteria. To exclude duplicates from the study search, results from all search terms were combined and PMIDs (unique identifier numbers used in PubMed) were used to exclude potential duplicates. The title and abstract of publications were carefully inspected and where necessary studies were removed that contained data reported in previous publications.

Inclusion criteria for preclinical studies on NMDA-R antagonists were as follows: (1) ketamine, PCP, MK-801, and/or memantine administration, (2) in vivo studies, (3) subanesthetic dosage, and (4) EEG/LFP/EcoG recordings. For human ketamine studies, inclusion criteria were: (1) EEG/MEG recordings, (2) subanesthetic dosage, (3) no previous or current history of neuropsychological disorder/substance abuse, and (4) sample size of *n* = 10 or more participants.

Finally, inclusion criteria for EEG/MEG studies in ScZ patients were as follows: (1) EEG/MEG and (2) sample size of *n* = 10 or more patients. In addition, publications that consisted of reviews, meta-analyses, case studies, and case reports were also excluded from further analysis. Preclinical and human studies were excluded where additional drugs in addition to NMDA-R antagonists were administered, such as psychotropic drugs. Finally, genetically modified animals were excluded.

The following parameters were retrieved: (1) preclinical studies: number, type, gender and age of animals, auditory task type, recording and location, frequency range, and type of NMDA-R antagonist drug as well as dosage; (2) healthy human studies: number, gender and age of participants, task type, imaging technique, frequency range, and pharmacological administration; (3) patient studies: patient type (CHR-P, FEP vs chronic ScZ), age and gender of participants, mean illness duration, medication status, task type, imaging technique, and frequency range.

### Statistical Analysis

For the estimation of effect sizes, the Comprehensive Meta-Analysis (CMA) software version 3.3.070 was used. Hedges’ *g* was calculated based on mean scores of gamma-band power values. When these values were not reported, exact *F*, *t*, or *P* values were employed. When effect sizes or appropriate statistics were missing from the reviewed articles, the authors were contacted to request the relevant data.

Effect size analysis was conducted on evoked gamma-band activity in ScZ patients and early-stage psychosis studies and data were available in 39 studies. We used R to plot the standardized mean differences with 95% confidence intervals. Funnel plots were visually inspected for symmetry to assess publication bias and outliers. Egger’s regression test, performed in R was used to assess potential asymmetry and interpreted.^46,47^

### Gamma-Band Measures

We examined the following measures of gamma-band (30–120 Hz) activity across studies: (1) Evoked power: Evoked activity occurs at a consistent time lag after the onset of an external event and can be identified by averaging the responses over several trials. Because of the close relationship with stimulus-onset, evoked oscillatory activity may reflect the encoding of stimulus information. (2) Intertrial phase coherence (ITPC): Assesses the phase modulations across trials.^48^ An ITPC value close to 0 reflects high variability of phase angles across epochs, whereas an ITPC value of 1 reflects all epochs having the same phase angle. (3) Induced oscillatory power: Induced oscillations are not locked to the onset of a stimulus. Analysis of induced oscillations must therefore be performed on a single-trial basis because averaging across trials would cancel out oscillations owing to random phase shifts.^48^

In addition, we included the analysis of baseline power where such parameters were available based on findings that NMDA-R antagonists increase baseline gamma-band power which in turn could impact task-related gamma-band oscillations.^44,49^

### Experimental Auditory Tasks

We included auditory tasks that are commonly employed in both preclinical and human studies, such as ASSRs, oddball- or pre-pulse inhibition (PPI) paradigms. However, only reports were considered that analyzed gamma-band responses to tones or stimulus trains. Studies that reported relative measures, such as the gamma-band band response as an expression of PPI or Mismatch Negativity (MMN), were not considered. Moreover, studies that used speech stimuli were not included.

### Risk of Bias

The risk of bias and quality of studies were assessed following Cochrane risk of bias guidelines^47^ for human randomized controlled studies, SYRCLE’s risk of bias tool for animal studies^50^ and the ROBINS-I assessment tool^51^ for matched cohort studies in ScZ patients (see supplementary tables S1–S3).

## Results

### Study Selection

**Fig. 1.**
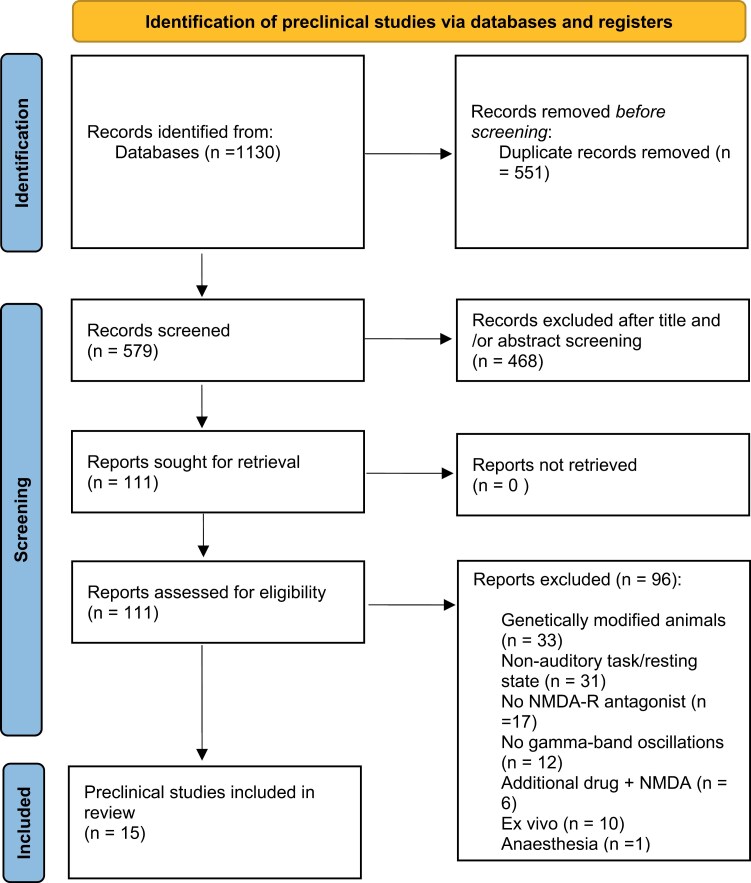
PRISMA chart of preclinical studies.

1130 preclinical studies were identified. After duplicate removal (*n* = 551), 468 reports were excluded after title or abstract screening. The remaining studies (*n* = 111) were assessed for eligibility and 96 were excluded, leading to 15 studies (see figure 1, supplementary table S4).

**Fig. 2.**
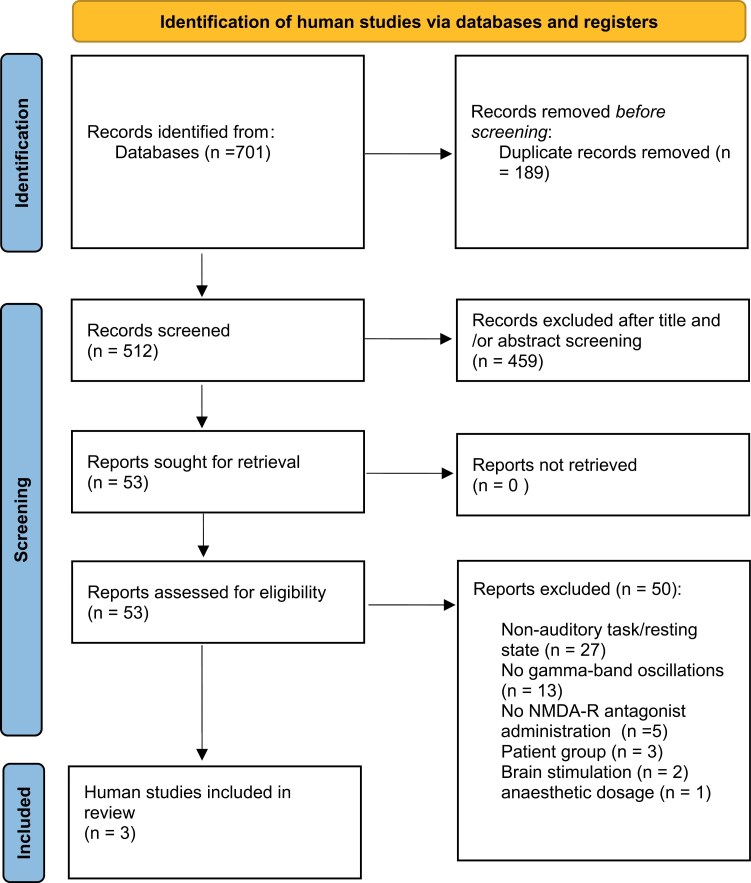
PRISMA chart of human pharmacological studies.

For studies in human participants with ketamine, *n* = 701 records were identified and *n* = 189 duplicates were removed. Following title and/or abstract screening *n* = 459 records were further excluded. Of the remaining 53 studies, 50 studies were excluded after full-text inspection, leading to a final sample consisting of 3 studies (see figure 2, supplementary table S5).

**Fig. 3.**
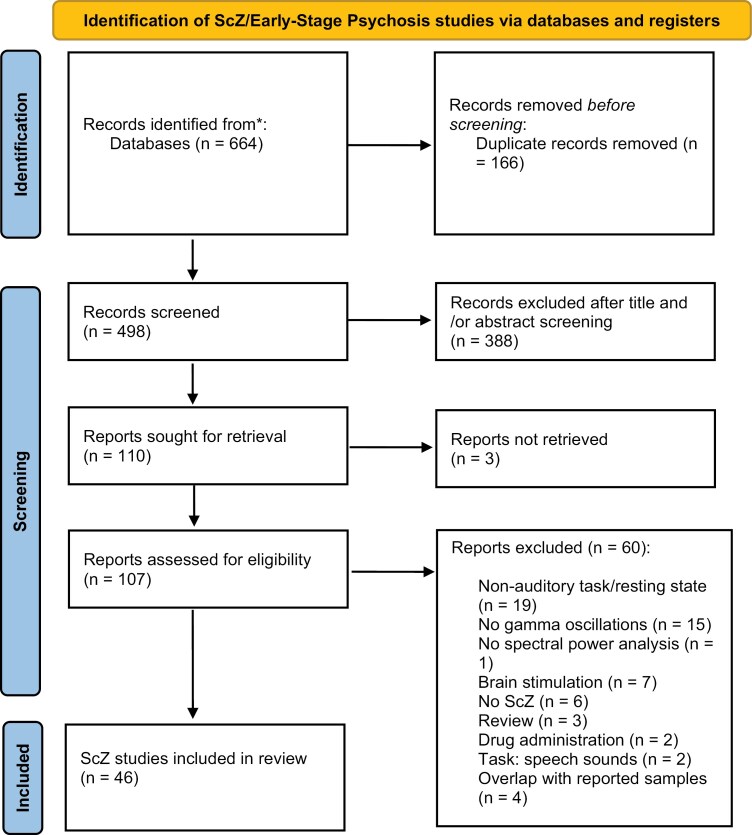
PRISMA Chart of EEG/MEG-Studies in Schizophrenia and Early-Stage Psychosis.

ScZ studies included 661 records, of which 166 were excluded after duplicate removal. Of the identified studies, 21 were obtained from a meta-analysis on ASSRs^35^ and titles and/or abstracts were screened for 387 studies. Full-text inspection of the reports assessed for eligibility (*n* = 105 studies) resulted in the further exclusion of 52 studies. Moreover, the papers by Maharajh et al,^52^ Roach et al,^53^ and Mulert et al^54^ were excluded due to the fact that these were either reanalyses of datasets from previously published studies or that clinical samples were partially overlapping with previous publications.^55^ The final sample consisted of 46 studies (see figure 3, supplementary table S6).

### Study Characteristics: Preclinical Studies

Twelve studies examined NMDA-R antagonists in rats and 3 studies in mice (supplementary material S4). A variety of auditory tasks were employed: 8 studies used ASSR paradigms, 4 studies employed an auditory oddball paradigm, 2 studies used a PPI, and 2 studies employed a paired-click paradigm.^56^ Pharmacological administration included ketamine (*n* = 9), 3 studies used MK-801 and 4 studies PCP. Two studies included both ketamine and MK-801.^57,58^ Twelve studies examined acute continuous dosages of NMDA-R antagonists, while *n* = 5 studies used chronic NMDA-R administration. Four studies examined both acute and chronic doses.

The majority of studies employed EEG recordings (13 studies) and *n* = 1 study utilized EcoG.^57^ EEG recordings included placement of electrodes over different cortical areas, including frontal regions (*n* = 3 studies), parietal lobe (*n* = 3 studies), temporal regions (*n* = 6 studies), occipital areas (*n* = 2 studies), and motor cortex (*n* = 1 study).^59^ Subcortical recordings included the hippocampus (*n* = 2 studies) and the cerebellum (*n* = 1 study).

All studies examined evoked spectral power (*n* = 14), while *n* = 5 also examined ITPC. We differentiated between low (30–60 Hz) (*n* = 14) and high gamma-band activity (60–120 Hz) (*n* = 10 studies). The majority of studies investigated evoked activity (*n* = 10). Six studies investigated induced gamma-band activity, with 2 studies examining both evoked and induced power.^60,61^ There were 6 studies that investigated baseline spectral power.

### Study Characteristics: Healthy Human Studies

*N* = 3 studies administered a bolus of ketamine followed by continuous, subanesthetic infusion (0.006 mg/kg/min) (see supplementary table S4). Auditory paradigms included auditory reaction tasks, (*n* = 2) and a paired-click paradigm (*n* = 1). All studies used EEG. Spectral power analysis was conducted by Hong et al^62^ while 2 studies investigated PLF and 1 investigated both power and PLF.^63^ All studies measured evoked activity and baseline spectral power.^64^

### Study Characteristics: ScZ Studies

Thirty studies examined patients with ScZ, while *n* = 4 studies focused on patients with FEP. One study^65^ investigated only CHR-P individuals (supplementary table S6). The remaining studies (*n* = 11) examined patient cohorts at different illness stages (CHR-P, FEP, and ScZ), including *n* = 5 studies with CHR-Ps. Only 1 study included antipsychotic naive FEP patients.^38^

The majority of studies employed ASSR paradigms (*n* = 29), while 10 studies used an auditory oddball task. Three studies investigated gamma-band activity during an auditory reaction task, and 4 studies during a paired-click paradigm. Thirty-four studies employed EEG while MEG was used in *n* = 11 studies. ITPC was examined in 32 studies, while evoked power was investigated in 40 studies and induced power in 8 studies. Of the 40 studies investigating evoked power, *n* = 2 studies^66,67^ reported total power and *n* = 5 reported event-related spectral perturbation. Finally, baseline activity was investigated in 6 studies.

### Risk of Bias Within and Across Studies

#### Preclinical NMDA-R Antagonist Studies

Only 1 study had a high overall risk of bias,^68^ while 4 studies presented unclear overall risk of bias^69–72^ and the remaining 10 studies had low overall risk. Random sequence allocation was high in 1 study^68^ and 3 studies were at unclear risk of bias.^68,70,71^ Random housing was considered at unclear risk of bias in 2 studies.^56,72^ Random outcome assessment was unclear in 3 studies and at high risk in 1 study^68^ (see supplementary table S1).

#### Human Ketamine Studies

Three studies were considered to have a low risk of bias. The blinding of outcome assessment was unclear in 1 study^62^ (see supplementary table S2).

#### ScZ Auditory Task Studies

Seven studies were considered to have a high overall risk of bias, 3 studies were at unclear overall risk and 42 studies presented with a low risk of bias (see supplementary table S3). High risk was detected in 6 studies concerning confounders and in 3 studies concerning selection bias,^73–75^ 1 study for attrition bias.^55^ Unclear risk was observed in 4 studies for confounding,^55,74,76^ 4 studies concerning selection bias,^66,74,77^ 1 study for misclassification bias^39^ and 1 study for measurement bias.^78^

#### Risk of Publication Bias

Egger’s regression test was significant (*t* = 3.65, df = 37, *P* ≤ .0001), suggesting the presence of publication bias. Moreover, a funnel plot indicated some asymmetry, suggesting that the studies investigated in the meta-analysis could be affected by reporting bias (supplementary figure S1).

Additionally, we used the “trim and fill” method to estimate the number of studies needed for the funnel plot to be symmetrical^79^ which indicates that the original results are overestimated due to publication bias (see supplementary figure S2).

### Preclinical Studies: Effects of NMDA-R Antagonists on Gamma-Band Activity

Evoked power: Eight out of 14 studies reported reductions in evoked gamma-band power following the administration of NMDA-R antagonists. The remaining studies report enhanced (*n* = 3),^49,60,69^ no change^80^ as well as bidirectional findings (*n* = 2). Ahnaou et al^81^ showed that acute ketamine increased gamma-band activity to standard and deviant stimuli during an oddball paradigm, while chronic ketamine administration decreased high gamma-band activity.

ITPC: Five out of 9 studies showed reduced gamma-band ITPC following NMDA-R antagonists. In contrast, 3 studies reported elevated gamma-band ITPC. Sivarao et al^82^ demonstrated a dose-dependent effect of ketamine: gamma-band ITPC was initially increased at 1 mg/kg while 30 mg/kg resulted initially in a reduction of gamma-band ITPC, followed by an increase after 30 min intravenous administration.

Induced power: Only the study by Lazarewicz et al^49^ examined the effects of ketamine on induced power, reporting a reduction of 30–80 Hz activity.

Baseline activity: Changes in baseline activity following the administration of NMDA-R antagonists were examined in 6 studies. Five studies^57,59,70,80,83^ reported an increase while only the study by Lee et al^84^ showed no effect of PCP on baseline power.

### Human Studies: Effects of NMDA-R Antagonists on Gamma-Band Oscillations

Evoked power: Two studies exhibited a reduction in evoked gamma activity following ketamine administration,^63,85^ while 1 study^62^ observed an increase in evoked gamma-band power (see supplementary table S6).

ITPC: Two studies^63,85^ observed reduced gamma-band ITPC following ketamine administration.

Correlations with psychopathology: There was an association between auditory gamma-band activity and negative symptoms, but the direction of the effect differed across studies. Thus, there was evidence for a positive correlation between negative symptoms and reduced gamma-band oscillations^62^ while Haaf et al^85^ and Curic et al.^63^ reported a negative relationship between gamma-band power and negative symptoms.

### Gamma-Band Activity in ScZ and Early-Stage Psychosis

#### Gamma-Band Activity in ScZ

Evoked power: Out of 37 studies investigating evoked gamma-band power, 17 studies reported significant reductions. Conversely, 4 studies^86–88^ documented an increase in evoked gamma-band activity, all of which were conducted in ScZ patients. Moreover, 7 studies^32,40,41,77,89–91^ did not identify significant differences between ScZ patients and healthy controls, although Rass et al^92^ discerned a trend level. The remaining studies presented mixed results concerning specific frequencies: Brenner et al,^66^ Puvvada et al,^93^ and Vierling-Claassen et al^67^ detected reductions toward 40 Hz ASSRs but not at other gamma-band frequencies.

ITPC: Twenty-four studies investigated ITPC of which 16 reported decreased gamma-band activity. Two studies^87,88^ reported increased ITPC whereas 3 did not observe group differences relative to healthy controls.^41,91,92^ Hirano et al^94^ reported no differences at 30 Hz but a significant reduction during 40 Hz ASSRs. Similar results were obtained by Tsuchimoto et al.^94^

Induced power: Seven studies compared induced power between ScZ-patient groups and healthy controls. One study detected a decrease^95^ while 3 reported an increase in induced power.^32,87,96^ The studies by Gallinat et al,^33^ Nguyen et al,^97^ and Popov et al^98^ found no differences in induced gamma-band power.

Baseline activity: Three studies^32,75,99^ reported an increase in baseline activity in ScZ patients vs healthy controls while 3 studies^39,73,88^ found no differences.

#### Effect Size Evoked Gamma-Band Power

The average Hedges’ *g* random-effect size was −0.63 for evoked gamma-band power (see supplementary figure 3).

#### Gamma-Band Activity in Early-Stage Psychosis

Evoked power: Out of 8 studies that examined evoked power in CHR-P and FEP groups, 5 reported reduced gamma-band power. Two studies indicated no significant differences,^74,100^ although Perez et al^74^ reported a trend-level reduction.^74^ Tada et al^101^ showed a reduction at 40 Hz ASSRs in both FEP and CHR groups but not at 30 Hz stimulation frequencies.

ITPC: Out of 9 studies, 6 detected reductions in ITPC while 2 studies^100,102^ found no difference albeit Leicht et al^102^ reported a trend-level reduction in CHR-Ps. Tada et al^101^ reported reduced ITPC at 40 Hz but not 30 Hz ASSRs in both FEP and CHR-Ps.

Induced power: Wang et al^103^ detected a reduction at 40 Hz power in FEPs.

#### Gamma-Band Activity and Symptom Correlations

The relationship between clinical symptoms and gamma-band oscillations in ScZ and early-stage psychosis was investigated in 26 studies. Fourteen reported no association between auditory gamma-band activity and psychopathology, while 9 studies observed positive and negative correlations with positive symptoms. Five studies observed negative correlations between reduced gamma-band activity and negative symptoms.^37,39,95,104,105^ In addition, 2 studies^101,106^ found a correlation with the Positive and Negative Syndrome Scale (PANSS) total scores while Fujimoto et al^107^ reported a correlation between gamma-band activity and disorganized symptoms.

## Discussion

The systematic review investigated the effects of NMDA-R antagonists on gamma-band activity during auditory stimulation in animal models and human electrophysiological recordings with the aim to compare these changes with the pattern of gamma-band deficits observed in ScZ and early-stage psychosis patients. It was hypothesized that NMDA-R hypofunction would recreate the pattern of gamma-band activity observed in clinical populations, given the extensive evidence for similarities in psychopathology between the effects of NMDA-R hypofunctioning in healthy volunteers and symptoms of ScZ^14^ as well as the potential overlap in circuit dysfunctions elicited by NMDA-R antagonists and observations in ScZ and early-stage psychosis.^44,108^

Specifically, we expected NMDA-R antagonists would lead to a deficit in the generation of gamma-band activity characterized by changes either to the signal-to-noise ratio^44^ or impaired generation of high-frequency activity.^32^ Crucially, we expected that NMDA-R administration would result in changes compatible with task-related deficits observed in ScZ patients and circuit deficits that may differ across illness stages.^38,91,109^

Overall, we could partially support these hypotheses. The majority of preclinical studies involving NMDA-R antagonists as well as findings in ScZ and early-stage psychosis support a reduction in evoked and ITPC gamma-band activity. Evoked gamma-band power and ITPC occur typically in a latency range of 50–150 ms, suggesting a role in the initial sensory registration and feedforward propagation of stimulus information.^110^ However, there was also evidence for increased gamma-band activity and biphasic effects in both patient^86,111^ and preclinical studies.^69,112^

In regard to induced gamma-band activity, the pattern of deficits was more variable, especially in patient populations where there was evidence for both up- and downregulated gamma-band activity. In contrast to evoked oscillatory, induced gamma-band oscillations are non-phased locked and tend to reflect cognitive processes, such as attention and memory.^113^

Finally, while NMDA-R antagonists were consistently associated with an increase in baseline activity in preclinical studies, the evidence from studies in early-stage psychosis and ScZ was inconclusive, suggesting that the mechanisms through which NMDA-R hypofunctioning impacts on aberrant gamma-band oscillations and circuit deficits may be different from those observed in clinical populations. However, further studies are required in ScZ and/or early-stage psychosis populations to substantiate this conclusion.

Previous studies^32,44^ have suggested that impairments in sensory-related gamma-band activity in ScZ are the result of an SNR deficit whereby elevated, ongoing gamma-band activity interferes with the generation of precisely timed neuronal oscillations. These data are consistent with findings from a previous systematic review of our group^25^ that examined the effects of NMDA-R antagonist administration on resting-state gamma-band activity. NMDA-R antagonists in preclinical and human studies were associated with an upregulation of high-frequency power while this observation was not consistent, however, with evidence from ScZ and early-stage psychosis cohorts.

There was considerable heterogeneity in pharmacological agents, recording and analysis techniques as well as experimental tasks that could have contributed toward differences in the direction of effects across studies. Of relevance is the contribution of different dosages of NMDA-R antagonists. While in human EEG/MEG data, studies used consistently subanesthetic dosages,^62,63,85^ only preclinical studies were able to examine the effects of different administration regimes and dosages of NMDA-R antagonists.

Previous studies had indicated that acute vs chronic administration of NMDA-R antagonists was associated with differences in both E/I-balance parameters^114^ and behavioral deficits.^17^ Consistent with these findings, Ahnaou et al^81^ found that acute ketamine administration increased gamma-band activity while chronic application reduced gamma-band oscillations. However, this was not confirmed by Martin et al.^61^ In addition, Sivarao et al^82^ showed a transient augmentation of gamma-band activity through low doses of ketamine (1 mg/kg) in mice while a high dose of ketamine (30 mg/kg) produced a sustained suppression of gamma-band activity in a 40-Hz ASSRs paradigm.

In ScZ patients, there was consistent evidence for a reduction in ITPC and evoked gamma-band activity although this was not confirmed in all studies, with evidence for both an absence of group differences^90^ as well as for increased gamma-band activity.^88^ Notably, a similar pattern was observed in FEP and CHR-P populations, suggesting that deficits in the generation of sensory-related gamma-band oscillations are present across illness stages and may have prognostic potential, for example, in predicting clinical outcomes in CHR-P populations.^38^ However, the number of studies in early-stage psychosis is still small, and further studies are needed to confirm these findings.

Across EEG/MEG studies in ScZ- and early-stage psychosis groups, there was also no consistent association with clinical symptoms. Changes in E/I balance that are mediated by gamma-band oscillations and glutamate levels have been proposed as a possible mechanism involved in the generation of auditory hallucinations^115,116^ and more recent evidence also suggests that ketamine induces both negative and positive symptoms in healthy volunteers.^117^

## Limitations

There are several limitations of the current systematic review. First, we compared several auditory paradigms across preclinical and human studies that may be associated with different corresponding gamma-band signatures. However, our review of ASSR studies in ScZ patients and early-stage psychosis (see supplementary analysis 1) suggests a similar pattern of deficits between ASSR studies and those employing other auditory paradigms. Moreover, both preclinical and studies in ScZ patients and early-stage psychosis measured gamma-band activity from different cortical and sometimes subcortical areas.

In addition, the presence of a publication bias in studies investigating auditory gamma-band activity in ScZ patients indicates that the reduction in gamma-band activity may have to be interpreted with caution. Furthermore, there is a limited number of studies that examined the differences and similarities between the acute and chronic effects of NMDA-R antagonists on auditory gamma-band oscillations. Current studies point to potential differences between acute and chronic dosages.^81,114^

Importantly, other neurotransmitters and receptor systems, such as dopamine and serotonin, are affected by NMDA-R antagonists, such as PCP and ketamine.^118,119^ Hence, aberrant auditory gamma-band activity may not be strictly generated from NMDA-Rs hypofunction, which could explain the mixed patterns emerging from the animal and human studies examined in the present systematic review.

The number of studies of human EEG/MEG recordings following ketamine administration was unfortunately small (*n* = 3) so no robust conclusions could be drawn. However, there was evidence for both increases^62^ as well as decreases^63,85^ of gamma-band activity across studies.

## Summary and Perspective

Our systematic review has highlighted that the administration of NMDA-R antagonists may have similar effects on auditory gamma-band activity as observed in ScZ and early-stage psychosis. However, the underlying mechanisms through which these changes occur may be different. While NMDA-R antagonists in preclinical studies lead to disinhibition of neural circuits, the associated increase of baseline gamma-band activity is not consistently observed in ScZ and early-stage psychosis patients. This conclusion is in line with the outcome of a recent systematic review,^25^ which compared changes in resting-state gamma-band activity following NMDA-R antagonists.

Thus, further research is required to identify the origin of circuit deficits in ScZ as well as the precise actions of NMDA-R antagonists on neural circuits and gamma-band activity. Recent work has, for example, indicated that ketamine causes spontaneously active neurons to become suppressed while previously silent neurons become spontaneously activated. This mechanism of action is mediated by the suppression of PV+ and somatostatin interneuron activity and inhibition of NMDA receptors and hyperpolarization-activated cyclic-nucleotide-gated channels.^120^ Accordingly, it is conceivable that a more complex pattern of GABAergic interneurons contributes toward the dysregulation of gamma-band oscillations following NMDA-R hypofunctioning which may be distinct from circuit deficits observed in ScZ.

## Supplementary Material

Supplementary material is available at https://academic.oup.com/schizophreniabulletin/.

sbae090_suppl_Supplementary_Material

## References

[1] Howes OD , Kapur S. The dopamine hypothesis of schizophrenia: version III—the final common pathway. Schizophr Bull. 2009;35(3):549–562. 19325164 10.1093/schbul/sbp006 PMC2669582

[2] Correll CU , Schooler NR. Negative symptoms in schizophrenia: a review and clinical guide for recognition, assessment, and treatment. Neuropsychiatr Dis Treat. 2020;16:519–534. 32110026 10.2147/NDT.S225643 PMC7041437

[3] Keefe RS , Bilder RM, Davis SM, et al.; CATIE Investigators. Neurocognitive effects of antipsychotic medications in patients with chronic schizophrenia in the CATIE Trial. Arch Gen Psychiatry. 2007;64(6):633–647. 17548746 10.1001/archpsyc.64.6.633

[4] Abi-Saab WM , D’Souza DC, Moghaddam B, Krystal JH. The NMDA antagonist model for schizophrenia: promise and pitfalls. Pharmacopsychiatry. 1998;31(suppl 2):104–109. 9754841 10.1055/s-2007-979354

[5] Coyle JT. NMDA receptor and schizophrenia: a brief history. Schizophr Bull. 2012;38(5):920–926. 22987850 10.1093/schbul/sbs076 PMC3446237

[6] Javitt DC , Kantrowitz JT. The glutamate/N-methyl-d-aspartate receptor (NMDAR) model of schizophrenia at 35: on the path from syndrome to disease. Schizophr Res. 2022;242:56–61. 35125283 10.1016/j.schres.2022.01.013

[7] McCutcheon RA , Krystal JH, Howes OD. Dopamine and glutamate in schizophrenia: biology, symptoms and treatment. World Psychiatry. 2020;19(1):15–33. 31922684 10.1002/wps.20693 PMC6953551

[8] Catts VS , Derminio DS, Hahn C-G, Weickert CS. Postsynaptic density levels of the NMDA receptor NR1 subunit and PSD-95 protein in prefrontal cortex from people with schizophrenia. npj Schizophr. 2015;1:15037. 27336043 10.1038/npjschz.2015.37 PMC4849460

[9] Akbarian S , Sucher NJ, Bradley D, et al. Selective alterations in gene expression for NMDA receptor subunits in prefrontal cortex of schizophrenics. J Neurosci. 1996;16(1):19–30. 8613785 10.1523/JNEUROSCI.16-01-00019.1996 PMC6578738

[10] Weickert CS , Fung SJ, Catts VS, et al. Molecular evidence of N-methyl-D-aspartate receptor hypofunction in schizophrenia. Mol Psychiatry. 2013;18(11):1185–1192. 23070074 10.1038/mp.2012.137 PMC3807670

[11] Woo TU , Walsh JP, Benes FM. Density of glutamic acid decarboxylase 67 messenger RNA-containing neurons that express the N-methyl-D-aspartate receptor subunit NR2A in the anterior cingulate cortex in schizophrenia and bipolar disorder. Arch Gen Psychiatry. 2004;61(7):649–657. 15237077 10.1001/archpsyc.61.7.649

[12] Pocklington AJ , Rees E, Walters JTR, et al. Novel findings from CNVS implicate inhibitory and excitatory signaling complexes in schizophrenia. Neuron. 2015;86(5):1203–1214. 26050040 10.1016/j.neuron.2015.04.022 PMC4460187

[13] Allen RM , Young SJ. Phencyclidine-induced psychosis. Am J Psychiatry. 1978;135(9):1081–1084. 696930 10.1176/ajp.135.9.1081

[14] Krystal JH , Karper LP, Seibyl JP, et al. Subanesthetic effects of the noncompetitive NMDA antagonist, ketamine, in humans. Psychotomimetic, perceptual, cognitive, and neuroendocrine responses. Arch Gen Psychiatry. 1994;51(3):199–214. 8122957 10.1001/archpsyc.1994.03950030035004

[15] Javitt DC , Zukin SR. Recent advances in the phencyclidine model of schizophrenia. Am J Psychiatry. 1991;148(10):1301–1308. 1654746 10.1176/ajp.148.10.1301

[16] Kantrowitz JT , Javitt DC. N-methyl-d-aspartate (NMDA) receptor dysfunction or dysregulation: the final common pathway on the road to schizophrenia? Brain Res Bull. 2010;83(3–4):108–121. 20417696 10.1016/j.brainresbull.2010.04.006 PMC2941541

[17] Phillips WA , Silverstein SM. Convergence of biological and psychological perspectives on cognitive coordination in schizophrenia. Behav Brain Sci. 2003;26(1):65–82; discussion 82–137. 14598440 10.1017/s0140525x03000025

[18] Romon T , Mengod G, Adell A. Expression of parvalbumin and glutamic acid decarboxylase-67 after acute administration of MK-801. Implications for the NMDA hypofunction model of schizophrenia. Psychopharmacology (Berl). 2011;217(2):231–238. 21465242 10.1007/s00213-011-2268-6

[19] Sohal VS , Zhang F, Yizhar O, Deisseroth K. Parvalbumin neurons and gamma rhythms enhance cortical circuit performance. Nature. 2009;459(7247):698–702. 19396159 10.1038/nature07991 PMC3969859

[20] Kopell N , LeMasson G. Rhythmogenesis, amplitude modulation, and multiplexing in a cortical architecture. Proc Natl Acad Sci U S A. 1994;91(22):10586–10590. 7937997 10.1073/pnas.91.22.10586 PMC45066

[21] Buzsaki G , Wang XJ. Mechanisms of gamma oscillations. Annu Rev Neurosci. 2012;35:203–225. 22443509 10.1146/annurev-neuro-062111-150444 PMC4049541

[22] Carlen M , Meletis K, Siegle JH, et al. A critical role for NMDA receptors in parvalbumin interneurons for gamma rhythm induction and behavior. Mol Psychiatry. 2012;17(5):537–548. 21468034 10.1038/mp.2011.31 PMC3335079

[23] Grent-‘t-Jong T , Gross J, Goense J, et al. Resting-state gamma-band power alterations in schizophrenia reveal E/I-balance abnormalities across illness-stages. Elife. 2018;e37799. 10.7554/eLife.37799 PMC6160226 30260771

[24] Rutter L , Carver FW, Holroyd T, et al. Magnetoencephalographic gamma power reduction in patients with schizophrenia during resting condition. Hum Brain Mapp. 2009;30(10):3254–3264. 19288463 10.1002/hbm.20746 PMC2748144

[25] Bianciardi B , Uhlhaas PJ. Do NMDA-R antagonists re-create patterns of spontaneous gamma-band activity in schizophrenia? A systematic review and perspective. Neurosci Biobehav Rev. 2021;124:308–323. 33581223 10.1016/j.neubiorev.2021.02.005

[26] Spencer KM , Salisbury DF, Shenton ME, McCarley RW. Gamma-band auditory steady-state responses are impaired in first episode psychosis. Biol Psychiatry. 2008;64(5):369–375. 18400208 10.1016/j.biopsych.2008.02.021 PMC2579257

[27] Grutzner C , Wibral M, Sun L, et al. Deficits in high- (>60 Hz) gamma-band oscillations during visual processing in schizophrenia. Front Hum Neurosci. 2013;7:88. 23532620 10.3389/fnhum.2013.00088 PMC3607810

[28] Grent-‘t-Jong T , Rivolta D, Sauer A, et al. MEG-measured visually induced gamma-band oscillations in chronic schizophrenia: evidence for impaired generation of rhythmic activity in ventral stream regions. Schizophr Res. 2016;176(2–3): 177–185. 27349815 10.1016/j.schres.2016.06.003

[29] Metzner C , Zurowski B, Steuber V. The role of parvalbumin-positive interneurons in auditory steady-state response deficits in schizophrenia. Sci Rep. 2019;9(1):18525. 31811155 10.1038/s41598-019-53682-5 PMC6898379

[30] Curley AA , Lewis DA. Cortical basket cell dysfunction in schizophrenia. J Physiol. 2012;590(4):715–724. 22219337 10.1113/jphysiol.2011.224659 PMC3381305

[31] Rivolta D , Heidegger T, Scheller B, et al. Ketamine dysregulates the amplitude and connectivity of high-frequency oscillations in cortical-subcortical networks in humans: evidence from resting-state magnetoencephalography-recordings. Schizophr Bull. 2015;41(5):1105–1114. 25987642 10.1093/schbul/sbv051 PMC4535642

[32] Hirano Y , Oribe N, Kanba S, Onitsuka T, Nestor PG, Spencer KM. Spontaneous gamma activity in schizophrenia. JAMA Psychiatry. 2015;72(8):813–821. 25587799 10.1001/jamapsychiatry.2014.2642 PMC4768724

[33] Gallinat J , Winterer G, Herrmann CS, Senkowski D. Reduced oscillatory gamma-band responses in unmedicated schizophrenic patients indicate impaired frontal network processing. Clin Neurophysiol. 2004;115(8):1863–1874. 15261865 10.1016/j.clinph.2004.03.013

[34] Haig AR , Gordon E, De Pascalis V, Meares RA, Bahramali H, Harris A. Gamma activity in schizophrenia: evidence of impaired network binding? Clin Neurophysiol. 2000;111(8):1461–1468. 10904228 10.1016/s1388-2457(00)00347-3

[35] Thune H , Recasens M, Uhlhaas PJ. The 40-Hz auditory steady-state response in patients with schizophrenia: a meta-analysis. JAMA Psychiatry. 2016;73(11):1145–1153. 27732692 10.1001/jamapsychiatry.2016.2619

[36] Onitsuka T , Tsuchimoto R, Oribe N, Spencer KM, Hirano Y. Neuronal imbalance of excitation and inhibition in schizophrenia: a scoping review of gamma-band ASSR findings. Psychiatry Clin Neurosci. 2022;76(12):610–619. 36069299 10.1111/pcn.13472

[37] Leicht G , Andreou C, Polomac N, et al. Reduced auditory evoked gamma band response and cognitive processing deficits in first episode schizophrenia. World J Biol Psychiatry. 2015;16:387–397. 25774562 10.3109/15622975.2015.1017605

[38] Grent-‘t-Jong T , Gajwani R, Gross J, et al. 40-Hz auditory steady-state responses characterize circuit dysfunctions and predict clinical outcomes in clinical high-risk for psychosis participants: a magnetoencephalography study. Biol Psychiatry. 2021;90(6):419–429. 34116790 10.1016/j.biopsych.2021.03.018

[39] Hamm JP , Gilmore CS, Picchetti NAM, Sponheim SR, Clementz BA. Abnormalities of neuronal oscillations and temporal integration to low- and high-frequency auditory stimulation in schizophrenia. Biol Psychiatry. 2011;69(10):989–996. 21216392 10.1016/j.biopsych.2010.11.021 PMC3174270

[40] Blumenfeld LD , Clementz BA. Response to the first stimulus determines reduced auditory evoked response suppression in schizophrenia: single trials analysis using MEG. Clin Neurophysiol. 2001;112:1650–1659. 11514248 10.1016/s1388-2457(01)00604-6

[41] Brockhaus-Dumke A , Mueller R, Faigle U, Klosterkoetter J. Sensory gating revisited: relation between brain oscillations and auditory evoked potentials in schizophrenia. Schizophr Res. 2008;99:238–249. 18160261 10.1016/j.schres.2007.10.034

[42] Hamm JP , Gilmore CS, Clementz BA. Augmented gamma band auditory steady-state responses: support for NMDA hypofunction in schizophrenia. Schizophr Res. 2012;138(1):1–7. 22542616 10.1016/j.schres.2012.04.003 PMC3601795

[43] Krystal JH , Anticevic A. Toward illness phase-specific pharmacotherapy for schizophrenia. Biol Psychiatry. 2015;78(11):738–740. 26542740 10.1016/j.biopsych.2015.08.017

[44] Saunders JA , Gandal MJ, Siegel SJ. NMDA antagonists recreate signal-to-noise ratio and timing perturbations present in schizophrenia. Neurobiol Dis. 2012;46(1):93–100. 22245663 10.1016/j.nbd.2011.12.049 PMC4161042

[45] Jadi MP , Behrens MM, Sejnowski TJ. Abnormal gamma oscillations in N-methyl-D-aspartate receptor hypofunction models of schizophrenia. Biol Psychiatry. 2016;79(9):716–726. 26281716 10.1016/j.biopsych.2015.07.005 PMC4720598

[46] Sterne JA , Davey Smith G. Sifting the evidence—what’s wrong with significance tests? BMJ. 2001;322(7280):226–231. 11159626 10.1136/bmj.322.7280.226 PMC1119478

[47] Higgins JP , Altman DG, Gøtzsche PC, et al.; Cochrane Bias Methods Group. The Cochrane Collaboration’s tool for assessing risk of bias in randomised trials. BMJ. 2011;343:d5928. 22008217 10.1136/bmj.d5928 PMC3196245

[48] Tallon-Baudry C , Bertrand O, Delpuech C, Pernier J. Stimulus specificity of phase-locked and non-phase-locked 40 Hz visual responses in human. J Neurosci. 1996;16(13):4240–4249. 8753885 10.1523/JNEUROSCI.16-13-04240.1996 PMC6579008

[49] Lazarewicz MT , Ehrlichman RS, Maxwell CR, Gandal MJ, Finkel LH, Siegel SJ. Ketamine modulates theta and gamma oscillations. J Cogn Neurosci. 2010;22(7):1452–1464. 19583475 10.1162/jocn.2009.21305

[50] Hooijmans CR , Rovers MM, de Vries RBM, Leenaars M, Ritskes-Hoitinga M, Langendam MW. SYRCLE’s risk of bias tool for animal studies. BMC Med Res Methodol. 2014;14:43. 24667063 10.1186/1471-2288-14-43 PMC4230647

[51] Sterne JA , Hernán MA, Reeves BC, et al. ROBINS-I: a tool for assessing risk of bias in non-randomised studies of interventions. BMJ. 2016;355:i4919. 27733354 10.1136/bmj.i4919 PMC5062054

[52] Maharajh K , Teale P, Rojas DC, Reite ML. Fluctuation of gamma-band phase synchronization within the auditory cortex in schizophrenia. Clin Neurophysiol. 2010;121(4):542–548. 20071232 10.1016/j.clinph.2009.12.010 PMC2834841

[53] Roach BJ , Ford JM, Mathalon DH. Gamma band phase delay in schizophrenia. Biol Psychiatry Cogn Neurosci Neuroimaging. 2019;4(2):131–139. 30314905 10.1016/j.bpsc.2018.08.011

[54] Mulert C , Kirsch V, Pascual-Marqui R, McCarley RW, Spencer KM. Long-range synchrony of gamma oscillations and auditory hallucination symptoms in schizophrenia. Int J Psychophysiol. 2011;79(1):55–63. 20713096 10.1016/j.ijpsycho.2010.08.004 PMC3017735

[55] Koshiyama D , Kirihara K, Tada M, et al. Auditory gamma oscillations predict global symptomatic outcome in the early stages of psychosis: a longitudinal investigation. Clin Neurophysiol. 2018;129(11):2268–2275. 30216911 10.1016/j.clinph.2018.08.007

[56] Raza MU , Sivarao DV. Test-retest reliability of tone-and 40 Hz train-evoked gamma oscillations in female rats and their sensitivity to low-dose NMDA channel blockade. Psychopharmacology (Berl). 2021;238:2325–2334. 33944972 10.1007/s00213-021-05856-1

[57] Jones NC , Anderson P, Rind G, Sullivan C, van den Buuse M, O’Brien TJ. Effects of aberrant gamma frequency oscillations on prepulse inhibition. Int J Neuropsychopharmacol. 2014;17:1671–1681. 24832766 10.1017/S1461145714000492

[58] Ma J , Leung LS. The supramammillo-septal-hippocampal pathway mediates sensorimotor gating impairment and hyperlocomotion induced by MK-801 and ketamine in rats. Psychopharmacology (Berl). 2007;191:961–974. 17219218 10.1007/s00213-006-0667-x

[59] Jones NC , Hudson M, Foreman J, et al. Brain-derived neurotrophic factor haploinsufficiency impairs high-frequency cortical oscillations in mice. Eur J Neurosci. 2018;48:2816–2825. 28925523 10.1111/ejn.13722

[60] Leishman E , O’Donnell BF, Millward JB, et al. Phencyclidine disrupts the auditory steady state response in rats. PLoS One. 2015;10:e0134979. 26258486 10.1371/journal.pone.0134979 PMC4530939

[61] Martin AMS , O’Donnell BF, Millward JB, et al. Acute phencyclidine alters neural oscillations evoked by tones in the auditory cortex of rats. Neuropsychobiology. 2017;75:53–62. 29065422 10.1159/000480511 PMC5752597

[62] Hong LE , Summerfelt A, Buchanan RW, et al. Gamma and delta neural oscillations and association with clinical symptoms under subanesthetic ketamine. Neuropsychopharmacology. 2010;35:632–640. 19890262 10.1038/npp.2009.168 PMC3055615

[63] Curic S , Leicht G, Thiebes S, et al. Reduced auditory evoked gamma-band response and schizophrenia-like clinical symptoms under subanesthetic ketamine. Neuropsychopharmacology. 2019;44:1239–1246. 30758327 10.1038/s41386-019-0328-5 PMC6785009

[64] Thiebes S , Steinmann S, Curic S, et al. Alterations in interhemispheric gamma-band connectivity are related to the emergence of auditory verbal hallucinations in healthy subjects during NMDA-receptor blockade. Neuropsychopharmacology. 2018;43:1608–1615. 29453445 10.1038/s41386-018-0014-z PMC5983549

[65] Leicht G , Vauth S, Polomac N, et al. EEG-informed fMRI reveals a disturbed gamma-band-specific network in subjects at high risk for psychosis. Schizophr Bull. 2016;42:239–249. 26163477 10.1093/schbul/sbv092 PMC4681551

[66] Brenner CA , Sporns O, Lysaker PH, O’Donnell BF. EEG synchronization to modulated auditory tones in schizophrenia, schizoaffective disorder, and schizotypal personality disorder. Am J Psychiatry. 2003;160:2238–2240. 14638599 10.1176/appi.ajp.160.12.2238

[67] Vierling-Claassen D , Siekmeier P, Stufflebeam S, Kopell N. Modeling GABA alterations in schizophrenia: a link between impaired inhibition and altered gamma and beta range auditory entrainment. J Neurophysiol. 2008;99(5):2656–2671. 18287555 10.1152/jn.00870.2007 PMC2679675

[68] Raith H , Schuelert N, Duveau V, et al. Differential effects of traxoprodil and S-ketamine on quantitative EEG and auditory event-related potentials as translational biomarkers in preclinical trials in rats and mice. Neuropharmacology. 2020;171:108072. 32243874 10.1016/j.neuropharm.2020.108072

[69] Ahnaou A , Biermans R, Drinkenburg WH. Modulation of mGlu2 receptors, but not PDE10A inhibition normalizes pharmacologically-induced deviance in auditory evoked potentials and oscillations in conscious rats. PLoS One. 2016;11:e0147365. 26808689 10.1371/journal.pone.0147365 PMC4726622

[70] Kozono N , Honda S, Tada M, et al. Auditory steady state response; nature and utility as a translational science tool. Sci Rep. 2019;9:1–10. 31186500 10.1038/s41598-019-44936-3 PMC6560088

[71] Qi R , Li J, Wu X, Geng X, Chen N, Yu H. Effects of ketamine on basal gamma band oscillation and sensory gating in prefrontal cortex of awake rats. Neurosci Bull. 2018;34:457–464. 29380249 10.1007/s12264-018-0208-8 PMC5960446

[72] Sullivan EM , Timi P, Hong LE, O’Donnell P. Effects of NMDA and GABA-A receptor antagonism on auditory steady-state synchronization in awake behaving rats,. Int J Neuropsychopharmacol. 2015;18:pyu118. 25556198 10.1093/ijnp/pyu118 PMC4540097

[73] Wilson TW , Hernandez OO, Asherin RM, Teale PD, Reite ML, Rojas DC. Cortical gamma generators suggest abnormal auditory circuitry in early-onset psychosis. Cereb Cortex. 2008;18:371–378. 17557901 10.1093/cercor/bhm062 PMC2648842

[74] Perez VB , Roach BJ, Woods SW, et al. Early auditory gamma-band responses in patients at clinical high risk for schizophrenia. Suppl Clin Neurophysiol. 2013;62:147–162. 24053038 10.1016/b978-0-7020-5307-8.00010-7 PMC4120874

[75] Parker DA , Hamm JP, McDowell JE, et al. Auditory steady-state EEG response across the schizo-bipolar spectrum. Schizophr Res. 2019;209:218–226. 31080153 10.1016/j.schres.2019.04.014 PMC6661193

[76] Hamm JP , Gilmore CS, Picchetti NAM, Sponheim SR, Clementz BA. Abnormalities of neuronal oscillations and temporal integration to low- and high-frequency auditory stimulation in schizophrenia. Biol Psychiatry. 2011;69: 989–996. 21216392 10.1016/j.biopsych.2010.11.021 PMC3174270

[77] Kirihara K , Rissling AJ, Swerdlow NR, Braff DL, Light GA. Hierarchical organization of gamma and theta oscillatory dynamics in schizophrenia. Biol Psychiatry. 2012;71(10): 873–880. 22361076 10.1016/j.biopsych.2012.01.016 PMC3434875

[78] Light GA , Hsu JL, Hsieh MH, et al. Gamma band oscillations reveal neural network cortical coherence dysfunction in schizophrenia patients. Biol Psychiatry. 2006;60:1231–1240. 16893524 10.1016/j.biopsych.2006.03.055

[79] Duval S , Tweedie R. Trim and fill: a simple funnel-plot-based method of testing and adjusting for publication bias in meta-analysis. Biometrics. 2000;56(2):455–463. 10877304 10.1111/j.0006-341x.2000.00455.x

[80] Ehrlichman RS , Gandal MJ, Maxwell CR, et al. N-methyl-d-aspartic acid receptor antagonist-induced frequency oscillations in mice recreate pattern of electrophysiological deficits in schizophrenia. Neuroscience. 2009;158(2):705–712. 19015010 10.1016/j.neuroscience.2008.10.031

[81] Ahnaou A , Huysmans H, Biermans R, Manyakov NV, Drinkenburg WHIM. Ketamine: differential neurophysiological dynamics in functional networks in the rat brain. Transl Psychiatry. 2017;7:e1237. 28926001 10.1038/tp.2017.198 PMC5639243

[82] Sivarao DV , Chen P, Senapati A, et al. 40 Hz auditory steady-state response is a pharmacodynamic biomarker for cortical NMDA receptors. Neuropsychopharmacology. 2016;41:2232–2240. 26837462 10.1038/npp.2016.17 PMC4946051

[83] Schuelert N , Dorner-Ciossek C, Brendel M, Rosenbrock H. A comprehensive analysis of auditory event-related potentials and network oscillations in an NMDA receptor antagonist mouse model using a novel wireless recording technology. Physiol Rep. 2018;6(16):e13782. 30155997 10.14814/phy2.13782 PMC6113138

[84] Lee M , Balla A, Sershen H, Sehatpour P, Lakatos P, Javitt DC. Rodent mismatch negativity/theta neuro-oscillatory response as a translational neurophysiological biomarker for N-methyl-D-aspartate receptor-based new treatment development in schizophrenia. Neuropsychopharmacology. 2018;43:571–582. 28816240 10.1038/npp.2017.176 PMC5770758

[85] Haaf M , Curic S, Steinmann S, Rauh J, Leicht G, Mulert C. Glycine attenuates impairments of stimulus-evoked gamma oscillations in the ketamine model of schizophrenia. Neuroimage. 2022;251:119004. 35176492 10.1016/j.neuroimage.2022.119004

[86] Hayrynen LK , Hamm JP, Sponheim SR, Clementz BA. Frequency-specific disruptions of neuronal oscillations reveal aberrant auditory processing in schizophrenia. Psychophysiology. 2016;53:786–795. 26933842 10.1111/psyp.12635

[87] Hamm JP , Gilmore CS, Clementz BA. Augmented gamma band auditory steady-state responses: support for NMDA hypofunction in schizophrenia. Schizophr Res. 2012;138:1–7. 22542616 10.1016/j.schres.2012.04.003 PMC3601795

[88] Kim S , Jang S-K, Kim D-W, et al. Cortical volume and 40-Hz auditory-steady-state responses in patients with schizophrenia and healthy controls. Neuroimage Clin. 2019;22:101732. 30851675 10.1016/j.nicl.2019.101732 PMC6407311

[89] Basar-Eroglu C , Mathes B, Brand A, Schmiedt-Fehr C. Occipital gamma response to auditory stimulation in patients with schizophrenia. Int J Psychophysiol. 2011;79:3–8. 21056599 10.1016/j.ijpsycho.2010.10.011

[90] Hong LE , Summerfelt A, McMahon R, et al. Evoked gamma band synchronization and the liability for schizophrenia. Schizophr Res. 2004;70:293–302. 15329305 10.1016/j.schres.2003.12.011

[91] Spencer KM , Niznikiewicz MA, Shenton ME, McCarley RW. Sensory-evoked gamma oscillations in chronic schizophrenia. Biol Psychiatry. 2008;63:744–747. 18083143 10.1016/j.biopsych.2007.10.017 PMC2330275

[92] Rass O , Forsyth JK, Krishnan GP, et al. Auditory steady state response in the schizophrenia, first-degree relatives, and schizotypal personality disorder. Schizophr Res. 2012;136(1–3):143–149. 22285558 10.1016/j.schres.2012.01.003 PMC3298621

[93] Puvvada KC , Summerfelt A, Du X, et al. Delta vs gamma auditory steady state synchrony in schizophrenia. Schizophr Bull. 2018;44(2):378–387. 29036430 10.1093/schbul/sbx078 PMC5814801

[94] Tsuchimoto R , Kanba S, Hirano S, et al. Reduced high and low frequency gamma synchronization in patients with chronic schizophrenia. Schizophr Res. 2011;133(1–3):99–105. 21849245 10.1016/j.schres.2011.07.020

[95] Fujimoto T , Okumura E, Takeuchi K, et al. Dysfunctional cortical connectivity during the auditory oddball task in patients with schizophrenia. Open Neuroimaging J. 2013;7:15–26. 10.2174/1874440001307010015 PMC3636485 23750187

[96] Teale P , Collins D, Maharajh K, Rojas DC, Kronberg E, Reite M. Cortical source estimates of gamma band amplitude and phase are different in schizophrenia. Neuroimage. 2008;42:1481–1489. 18634887 10.1016/j.neuroimage.2008.06.020 PMC2637224

[97] Nguyen AT , Hetrick WP, O’Donnell BF, Brenner CA. Abnormal beta and gamma frequency neural oscillations mediate auditory sensory gating deficit in schizophrenia. J Psychiatr Res. 2020;124:13–21. 32109667 10.1016/j.jpsychires.2020.01.014

[98] Popov T , Jordanov T, Weisz N, Elbert T, Rockstroh B, Miller GA. Evoked and induced oscillatory activity contributes to abnormal auditory sensory gating in schizophrenia. Neuroimage. 2011;56:307–314. 21316464 10.1016/j.neuroimage.2011.02.016

[99] Edgar JC , Chen Y-H, Lanza M, et al. Cortical thickness as a contributor to abnormal oscillations in schizophrenia? Neuroimage Clin. 2014;4:122–129. 24371794 10.1016/j.nicl.2013.11.004 PMC3871288

[100] Oribe N , Hirano Y, Del Re E, et al. Progressive reduction of auditory evoked gamma in first episode schizophrenia but not clinical high risk individuals. Schizophr Res. 2019;208:145–152. 31005464 10.1016/j.schres.2019.03.025

[101] Tada M , Nagai T, Kirihara K, et al. Differential alterations of auditory gamma oscillatory responses between pre-onset high-risk individuals and first-episode schizophrenia. Cereb Cortex. 2016;26(3):1027–1035. 25452567 10.1093/cercor/bhu278

[102] Leicht G , Kirsch V, Giegling I, et al. Reduced early auditory evoked gamma-band response in patients with schizophrenia. Biol Psychiatry. 2010;67:224–231. 19765689 10.1016/j.biopsych.2009.07.033

[103] Wang J , Tang Y, Curtin A, et al. Abnormal auditory-evoked gamma band oscillations in first-episode schizophrenia during both eye open and eye close states. Prog Neuropsychopharmacol Biol Psychiatry. 2018;86:279–286. 29705712 10.1016/j.pnpbp.2018.04.016

[104] Griskova-Bulanova I , Hubl D, van Swam C, Dierks T, Koenig T. Early- and late-latency gamma auditory steady-state response in schizophrenia during closed eyes: does hallucination status matter? Clin Neurophysiol. 2016;127(5): 2214–2221. 27072092 10.1016/j.clinph.2016.02.009

[105] Koshiyama D , Miyakoshi M, Joshi YB, et al. A distributed frontotemporal network underlies gamma-band synchronization impairments in schizophrenia patients. Neuropsychopharmacology. 2020;45(13):2198–2206. 32829382 10.1038/s41386-020-00806-5 PMC7784692

[106] Taylor GW , McCarley RW, Salisbury DF. Early auditory gamma band response abnormalities in first hospitalized schizophrenia. Suppl Clin Neurophysiol. 2013;62:131–145. 24053037 10.1016/b978-0-7020-5307-8.00009-0 PMC5768311

[107] Fujimoto T , Okumura E, Takeuchi K, et al. Dysfunctional cortical connectivity during the auditory oddball task in patients with schizophrenia. *Open Neuroimag J*. 2013;7:15–26. 23750187 10.2174/1874440001307010015 PMC3636485

[108] Anticevic A , Corlett PR, Cole MW, et al. N-methyl-D-aspartate receptor antagonist effects on prefrontal cortical connectivity better model early than chronic schizophrenia. Biol Psychiatry. 2015;77(6):569–580. 25281999 10.1016/j.biopsych.2014.07.022

[109] Kwon JS , O’Donnell BF, Wallenstein GV, et al. Gamma frequency-range abnormalities to auditory stimulation in schizophrenia. Arch Gen Psychiatry. 1999;56(11):1001–1005. 10565499 10.1001/archpsyc.56.11.1001 PMC2863027

[110] Uhlhaas PJ , Singer W. Abnormal neural oscillations and synchrony in schizophrenia. Nat Rev Neurosci. 2010;11(2):100–113. 20087360 10.1038/nrn2774

[111] Hamm JP , Bobilev AM, Hayrynen LK, et al. Stimulus train duration but not attention moderates γ-band entrainment abnormalities in schizophrenia. Schizophr Res. 2015;165:97–102. 25868936 10.1016/j.schres.2015.02.016 PMC5538018

[112] Sivarao DV , Chen P, Senapati A, et al. 40 Hz auditory steady-state response is a pharmacodynamic biomarker for cortical NMDA receptors. Neuropsychopharmacology. 2016;41(9):2232–2240. 26837462 10.1038/npp.2016.17 PMC4946051

[113] Uhlhaas PJ , Singer W. What do disturbances in neural synchrony tell us about autism? Biol Psychiatry. 2007;62(3): 190–191. 17631116 10.1016/j.biopsych.2007.05.023

[114] McNally JM , McCarley RW, Brown RE. Chronic ketamine reduces the peak frequency of gamma oscillations in mouse prefrontal cortex ex vivo. Front Psychiatry. 2013;4:106. 24062700 10.3389/fpsyt.2013.00106 PMC3775128

[115] Hugdahl K , Craven AR, Nygård M, et al. Glutamate as a mediating transmitter for auditory hallucinations in schizophrenia: a (1)H MRS study. Schizophr Res. 2015;161(2–3): 252–260. 25542859 10.1016/j.schres.2014.11.015

[116] Spencer KM , Niznikiewicz MA, Nestor PG, Shenton ME, McCarley RW. Left auditory cortex gamma synchronization and auditory hallucination symptoms in schizophrenia. BMC Neurosci. 2009;10:85. 19619324 10.1186/1471-2202-10-85 PMC2719648

[117] Beck K , Hindley G, Borgan F, et al. Association of ketamine with psychiatric symptoms and implications for its therapeutic use and for understanding schizophrenia: a systematic review and meta-analysis. JAMA Netw Open. 2020;3(5):e204693. 32437573 10.1001/jamanetworkopen.2020.4693 PMC7243091

[118] Kokkinou M , Ashok AH, Howes OD. The effects of ketamine on dopaminergic function: meta-analysis and review of the implications for neuropsychiatric disorders. Mol Psychiatry. 2018;23(1):59–69. 28972576 10.1038/mp.2017.190 PMC5754467

[119] Kessler RC , Adler L, Ames M, et al. The World Health Organization Adult ADHD Self-Report Scale (ASRS): a short screening scale for use in the general population. Psychol Med. 2005;35(2):245–256. 15841682 10.1017/s0033291704002892

[120] Cichon J , Wasilczuk AZ, Looger LL, Contreras D, Kelz MB, Proekt A. Ketamine triggers a switch in excitatory neuronal activity across neocortex. Nat Neurosci. 2023;26(1):39–52. 36424433 10.1038/s41593-022-01203-5 PMC10823523

