# Correction to: Effects of *N*-Methyl-d-Aspartate Receptor Antagonists on Gamma-Band Activity During Auditory Stimulation Compared With Electro/Magneto-encephalographic Data in Schizophrenia and Early-Stage Psychosis: A Systematic Review and Perspective

**DOI:** 10.1093/schbul/sbaf082

**Published:** 2025-05-22

This is a correction to: Bianca Bianciardi, Helena Mastek, Michelle Franka, Peter J Uhlhaas, Effects of *N*-Methyl-d-Aspartate Receptor Antagonists on Gamma-Band Activity During Auditory Stimulation Compared With Electro/Magneto-encephalographic Data in Schizophrenia and Early-Stage Psychosis: A Systematic Review and Perspective, *Schizophrenia Bulletin*, Volume 50, Issue 5, September 2024, Pages 1104–1116, https://doi.org/10.1093/schbul/sbae090.

In the originally published version of this manuscript, an error occurred in the interpretation of the findings by Curic et al. (2019) and Haaf et al. (2022). Both papers used a different metric for reporting the correlation between negative symptoms and gamma-band activity, when in fact the actual relationship between changes in gamma-band power and negative symptoms was the same.

Accordingly, in the section ‘*Human Studies: Effects of NMDA-R Antagonists on Gamma-Band Oscillations’,* a sentence has been corrected from ‘Thus, there was evidence for a positive correlation between negative symptoms and reduced gamma-band oscillations^62,63^ while Haaf et al^85^ reported a negative relationship between gamma-band power and negative symptoms’ to ‘Thus, there was evidence for a positive correlation between negative symptoms and reduced gamma-band oscillations^62,^ while Haaf et al^85^ and Curic et al.^63^ reported a negative relationship between gamma-band power and negative symptoms.’

