# Supplementary material for: Effects of N-Methyl-d-Aspartate Receptor Antagonists on Gamma-Band Activity During Auditory Stimulation Compared With Electro/Magneto-encephalographic Data in Schizophrenia and Early-Stage Psychosis: A Systematic Review and Perspective
Source: Schizophr Bull. 2024 Jun 27;50(5):1104–16. doi: 10.1093/schbul/sbae090 (PMC11349021; doi:10.1093/schbul/sbae090)
Supplement: sbae090_suppl_Supplementary_Material [file sbae090_suppl_supplementary_material.zip › SI Analysis 1_6.6.docx]

**SI Material Analysis 1. 40 Hz ASSRs in Schizophrenia and Early-Stage Psychosis**

**1.Schizophrenia Patients**

Evoked power: Out of 26 studies that examined 40 Hz ASSR in schizophrenia patients, 23 examined evoked power. Fifteen studies detected a reduction in 40 Hz ASSR while four studies [1-4] reported an increase in evoked activity. The remaining studies found no differences in 40 Hz ASSRs.

ITPC: Eighteen studies examined ITPC during 40 Hz ASSRs of which fourteen identified a reduction in ITPC, while only two detected an increase [2, 3]. Rass [5] and Kim et al. [2] found no differences in 40 Hz ASSR ITPC-activity.

Induced activity: Three studies that examined induced activity reported increased gamma-band power [3, 6, 7].

Baseline activity: Two studies found increases in baseline activity [1, 8], whereas three studies [2, 3, 9] reported no differences.

**2. Early-Stage Psychosis**

Evoked activity: Three studies [10-12] examined evoked gamma-band power in CHR-Ps and FEP-groups. There was consistent evidence for a reduction during 40 Hz ASSRs.

ITPC: Similarly, ITPC analyses revealed a consistent reduction across studies [10, 12-14]. Induced activity: Wang et al. [14] reported a reduction in induced activity during 40 Hz ASSRs.

Baseline: There were no differences in baseline activity for both and FEP and CHR-P groups as reported by Grent-'T-Jong et al. [10].

**References**

1. Edgar, J.C., et al., *Cortical thickness as a contributor to abnormal oscillations in schizophrenia?*, in *NeuroImage: Clinical*. 2014, The Authors. p. 122-129.

2. Kim, S., et al., *Cortical volume and 40-Hz auditory-steady-state responses in patients with schizophrenia and healthy controls.* Neuroimage Clin, 2019. **22**: p. 101732.

3. Hamm, J.P., C.S. Gilmore, and B.A. Clementz, *Augmented gamma band auditory steady-state responses: support for NMDA hypofunction in schizophrenia.* Schizophr Res, 2012. **138**(1): p. 1-7.

4. Hayrynen, L.K., et al., *Frequency-specific disruptions of neuronal oscillations reveal aberrant auditory processing in schizophrenia*, in *Psychophysiology*. 2016. p. 786-795.

5. Rass, O., et al., *Auditory steady state response in the schizophrenia, first-degree relatives, and schizotypal personality disorder.* Schizophr Res, 2012. **136**(1-3): p. 143-9.

6. Hirano, Y., et al., *Spontaneous Gamma Activity in Schizophrenia.* JAMA Psychiatry, 2015. **72**(8): p. 813-21.

7. Teale, P., et al., *Cortical source estimates of gamma band amplitude and phase are different in schizophrenia*, in *NeuroImage*. 2008. p. 1481-1489.

8. Parker, D.A., et al., *Auditory steady-state EEG response across the schizo-bipolar spectrum*, in *Schizophrenia Research*. 2019, Elsevier B.V. p. 218-226.

9. Wilson, T.W., et al., *Cortical gamma generators suggest abnormal auditory circuitry in early-onset psychosis*, in *Cerebral Cortex*. 2008. p. 371-378.

10. Grent-'t-Jong, T., et al., *40-Hz Auditory Steady-State Responses Characterize Circuit Dysfunctions and Predict Clinical Outcomes in Clinical High-Risk for Psychosis Participants: A Magnetoencephalography Study.* Biol Psychiatry, 2021. **90**(6): p. 419-429.

11. Spencer, K.M., et al., *Gamma-band auditory steady-state responses are impaired in first episode psychosis.* Biol Psychiatry, 2008. **64**(5): p. 369-75.

12. Tada, M., et al., *Differential Alterations of Auditory Gamma Oscillatory Responses Between Pre-Onset High-Risk Individuals and First-Episode Schizophrenia.* Cereb Cortex, 2016. **26**(3): p. 1027-1035.

13. Spencer, K.M., et al., *γ-Band Auditory Steady-State Responses Are Impaired in First Episode Psychosis*, in *Biological Psychiatry*. 2008. p. 369-375.

14. Wang, J., et al., *Abnormal auditory-evoked gamma band oscillations in first-episode schizophrenia during both eye open and eye close states.* Prog Neuropsychopharmacol Biol Psychiatry, 2018. **86**: p. 279-286.
