# Supplementary material for: Effects of N-Methyl-d-Aspartate Receptor Antagonists on Gamma-Band Activity During Auditory Stimulation Compared With Electro/Magneto-encephalographic Data in Schizophrenia and Early-Stage Psychosis: A Systematic Review and Perspective
Source: Schizophr Bull. 2024 Jun 27;50(5):1104–16. doi: 10.1093/schbul/sbae090 (PMC11349021; doi:10.1093/schbul/sbae090)
Supplement: sbae090_suppl_Supplementary_Material [file sbae090_suppl_supplementary_material.zip › SI Table 1_Uhl_6.6.docx]

**SI Table 1. Risk of bias assessment of preclinical studies**

|  | **H** | High risk |  |  |  |  |  |  |  |  |
| --- | --- | --- | --- | --- | --- | --- | --- | --- | --- | --- |
|  | **L** | Low risk |  |  |  |  |  |  |  |  |
|  | **?** | Unknown |  |  |  |  |  |  |  |  |
|  | **N/A** |  |  |  |  |  |  |  |  |  |
|  |  |  |  |  |  |  |  |  |  |  |
|  | Random Sequence generation | | Baseline characteristics | Allocation concealment | Random housing | Blinding | Random outcome assessment | Incomplete outcome data | Selective reporting | Overall Bias |
| Ahnaou et al., 2016 | L | | L | L | L | L | L | L | L | L |
| Ahnaou et al., 2017 | ? | | L | L | L | L | ? | L | L | ? |
| Jones et al., 2014 | L | | N/A | L | L | L | L | L | L | L |
| Jones et al., 2018 | L | | N/A | L | L | L | L | L | L | L |
| Kozono et al, 2019 | ? | | N/A | L | L | L | ? | L | L | ? |
| Lee et al., 2018 | L | | L | L | L | L | L | L | L | L |
| Leishman et al., 2015 | L | | L | L | L | L | L | L | L | L |
| Martin et al., 2017 | L | | L | L | L | L | L | L | L | L |
| Raith et al., 2020 | H | | N/A | L | L | L | H | L | L | H |
| Raza et al., 2021 | L | | N/A | L | ? | L | L | L | L | L |
| Sivarao et al., 2016 | L | | N/A | L | L | L | L | L | L | L |
| Sullivan et al., 2015 | L | | N/A | L | ? | L | L | L | L | ? |
| Ehrlichman et al., 2009 | L | | N/A | L | ? | ? | ? | L | L | ? |
| Lazarewicz et al., 2009 | L | | N/A | L | L | L | L | L | L | L |
| Schuelert et al., 2018 | L | | N/A | L | L | L | L | L | L | L |
