# Supplementary material for: Effects of N-Methyl-d-Aspartate Receptor Antagonists on Gamma-Band Activity During Auditory Stimulation Compared With Electro/Magneto-encephalographic Data in Schizophrenia and Early-Stage Psychosis: A Systematic Review and Perspective
Source: Schizophr Bull. 2024 Jun 27;50(5):1104–16. doi: 10.1093/schbul/sbae090 (PMC11349021; doi:10.1093/schbul/sbae090)
Supplement: sbae090_suppl_Supplementary_Material [file sbae090_suppl_supplementary_material.zip › SI Table 2_Uhl_6.6.docx]

**SI Table 2. Risk of bias assessment of healthy human studies.**

| **H** | | High risk | |  |  |  |  |  |  |
| --- | --- | --- | --- | --- | --- | --- | --- | --- | --- |
| **L** | | Low risk | |  |  |  |  |  |  |
| **?** | | Unknown | |  |  |  |  |  |  |
|  | |  | |  |  |  |  |  |  |
|  | Random sequence generation | | Allocation concealment | | Blinding of participants and personnel | Blinding of outcome assessment | Incomplete outcome data | Selective reporting | Overall bias |
| Curic et al., 2019 | L | | L | | L | L | L | L | L |
| Haaf et al., 2021 | L | | L | | L | L | L | L | L |
| Hong et al., 2010 | L | | L | | L | ? | L | L | ? |
