# Supplementary material for: Effects of N-Methyl-d-Aspartate Receptor Antagonists on Gamma-Band Activity During Auditory Stimulation Compared With Electro/Magneto-encephalographic Data in Schizophrenia and Early-Stage Psychosis: A Systematic Review and Perspective
Source: Schizophr Bull. 2024 Jun 27;50(5):1104–16. doi: 10.1093/schbul/sbae090 (PMC11349021; doi:10.1093/schbul/sbae090)
Supplement: sbae090_suppl_Supplementary_Material [file sbae090_suppl_supplementary_material.zip › SI Table 3_Uhl_6.6.docx]

**SI Table 3. Risk of bias assessment of ScZ studies and Early-Stage Psychosis**

|  | **H** | High risk | |  |  |  |  |  |  |  |
| --- | --- | --- | --- | --- | --- | --- | --- | --- | --- | --- |
|  | **L** | Low risk | |  |  |  |  |  |  |  |
|  | **?** | Unknown | |  |  |  |  |  |  |  |
|  |  |  | |  |  |  |  |  |  |  |
|  | Confounding | | Selection bias | | Measurement bias | Performance bias | Attrition bias | Misclassification bias | Selective reporting | Overall |
| Kwon et al., 1999 | L | | L | | L | L | L | L | L | L |
| Brenner et al., 2003 | H | | ? | | L | L | L | L | L | H |
| Hong et al., 2004 | L | | L | | L | L | L | L | L | L |
| Spencer et al., 2008a | L | | L | | L | L | L | L | L | L |
| Vierling- Claassen et al, 2008 | L | | L | | L | L | L | L | L | L |
| Teale et al., 2008 | L | | L | | L | L | L | L | L | L |
| Wilson et al., 2008 | L | | H | | L | L | L | H | L | H |
| Krishnan et al, 2009 | L | | L | | L | L | L | L | L | L |
| Spencer et al, 2009 | L | | L | | L | L | ? | L | L | ? |
| Hamm et al., 2011 | ? | | L | | L | L | L | ? | L | ? |
| Tsuchimoto et al, 2011 | L | | L | | L | L | L | L | L | L |
| Rass et al, 2012 | L | | L | | L | L | L | L | L | L |
| Kirihara et al, 2012 | H | | ? | | L | L | L | L | L | H |
| Edgar et al., 2013 | L | | L | | L | L | L | L | L | L |
| Tada et al., 2016 | L | | L | | L | L | L | L | L | L |
| Hirano et al, 2015 | L | | L | | L | L | L | L | L | L |
| Hamm et al., 2015 | L | | L | | L | L | L | L | L | L |
| Hamm et al, 2012 | L | | L | | L | L | L | L | L | L |
| Parker et al., 2019 | H | | H | | L | L | L | L | L | H |
| Light et al., 2006 | H | | L | | ? | L | L | L | L | H |
| Roach et al., 2008 | L | | L | | L | L | L | L | L | L |
| Leicht et al., 2010 | L | | L | | L | L | L | L | L | L |
| Spencer et al., 2008.b | L | | L | | L | L | L | L | L | L |
| Brockhaus-Dumke et al., 2008 | L | | L | | L | L | L | L | L | L |
| Perez et al., 2013 | H | | H | | L | L | L | L | L | H |
| Leicht et al., 2016 | L | | L | | L | L | L | L | L | L |
| Puvvada et al., 2017 | L | | L | | L | L | L | L | L | L |
| Gallinat et al., 2004 | L | | L | | L | L | L | L | L | L |
| Koshiyama, et al., 2020 | L | | L | | L | L | L | L | L | L |
| Wang et al., 2018 | ? | | L | | L | L | L | L | L | L |
| Nguyen et al., 2020 | L | | L | | L | L | L | L | L | L |
| Leicht et al., 2015 | L | | L | | L | L | L | L | L | L |
| Fujimoto, et al., 2013 | L | | L | | L | L | L | L | L | L |
| Griskova-Bulanova et al.,  2016 | L | | L | | L | L | L | L | L | L |
| Basar-Eroglu et al., 2011 | L | | L | | L | L | L | L | L | L |
| Taylor et al., 2013 | L | | L | | L | L | L | L | L | L |
| Kim et al., 2019 | L | | L | | L | L | L | L | L | L |
| Blumenfeld & Clementz, 2001 | ? | | ? | | L | L | L | L | L | ? |
| Hayrynen et al., 2016 | L | | L | | L | L | L | L | L | L |
| Popov et al., 2011 | L | | L | | L | L | L | L | L | L |
| Grent-‘t-Jong, et al., 2021 | L | | L | | L | L | L | L | L | L |
| Oribe et al., 2019 | L | | L | | L | L | L | L | L | L |
| Khadimallah et al., 2019 | L | | L | | L | L | L | L | L | L |
| Hall et al., 2011 | H | | L | | L | L | L | L | L | H |
| Zhou et al., 2018 | L | | L | | L | L | L | L | L | L |
