# Supplementary material for: Effects of N-Methyl-d-Aspartate Receptor Antagonists on Gamma-Band Activity During Auditory Stimulation Compared With Electro/Magneto-encephalographic Data in Schizophrenia and Early-Stage Psychosis: A Systematic Review and Perspective
Source: Schizophr Bull. 2024 Jun 27;50(5):1104–16. doi: 10.1093/schbul/sbae090 (PMC11349021; doi:10.1093/schbul/sbae090)
Supplement: sbae090_suppl_Supplementary_Material [file sbae090_suppl_supplementary_material.zip › SI Table 4_Preclinical_Uhl_6.6.docx]

| SI Table 4. *Summary of Preclinical Studies* | | | | | | | |
| --- | --- | --- | --- | --- | --- | --- | --- |
| REFERENCE | Animals | Task | Recording/  Location | Analysis | Pharmacology | Study design | Main results |
| Ahnaou et al., 2016 | N = 16 male Sprague Dawley rats | Paired click paradigm | EEG at frontal, parietal and occipital sites (bilaterally). | Low (32-48 Hz) and high (52-100 Hz) evoked power in response to S1 and S2 . | Acute PCP (3 mg/kg) or vehicle | Between-subject design | PCP enhanced gamma-band power in response to both S1 and S2 stimuli. |
| Ahnaou et al., 2017 | N = 32 Sprague Dawley rats | Oddball paradigm | EEG at frontal, parietal and occipital sites (bilaterally) | Low (32-48 Hz) and high (52-100 Hz) power and ITPC in response to standard and deviant sounds. | Acute (2.5, 5 and 10 mg kg -1) and chronic ketamine (2.5, 5 and 10 mg kg -1) for 4 weeks | Between-subject design | Acute ketamine increased gamma-band power to standard and deviant stimuli, while chronic chronic ketamine decreased high gamma-band power and ITPC. |
| Ehrlichman et al. 2009 | N = 12 male B6 mice | ﻿White noise clicks | EEG-hippocampal  recording | 30-80 Hz  evoked and baseline power | Acute ﻿(20 mg/kg) ketamine | Between-subject design | ﻿Ketamine enhanced baseline power while evoked gamma-band power was not affected. |
| Jones et al., 2014 | N = 12 male, adult Wistar rats | PPI paradigm | ECoG cortical recording | Evoked (30-80 Hz) power to prepulse-stimulus and baseline activity | Acute Ketamine (5 mg/kg), MK-801 (0.16 mg/kg) or vehicle | Pseudo-randomised; Repeated-measure design | Ketamine and MK-801 increased baseline power while reducing evoked gamma-band power. |
| Jones et al., 2018 | N = 8 male Wild type mice | 20 ms sounds | EEG, electrodes above motor cortex | Evoked 30-80 Hz of the prepulse stimulus, baseline power | Acute MK-801 (0.3 mg/kg i.p.) or vehicle | Pseudo-randomised; Repeated-measure design | MK-801 elevated baseline but reduced evoked gamma-band power |
| Kozono et al, 2019 | N = 16 adult Sprague-Dawley rats | 40 Hz ASSR paradigm (click trains) | EEG – temporal and parietal cortices | 30-80 Hz ITPC, evoked and baseline power | Acute ketamine hydrochloride (30 mg/2 mL/kg, s.c.) or vehicle. | Repeated-measure design. | 40-70 Hz power was reduced following ketamine administration and increased after 70-100 mins. ITPC increased after ketamine administration at 40 Hz ASSR. Baseline gamma-band power was increased . |
| ﻿Lazarewicz et al. 2009 | N = 20 adult male B6 mice | Paired-click paradigm | EEG-hippocampal  recording | 30-80 Hz  Evoked and induced power to the 1st click | Acute ﻿(5 and 20 mg/kg ip) ketamine | Between-subject design | Ketamine enhanced both background and evoked power,but decreased induced power. |
| Lee et al., 2018 | N = 21 Sprague–Dawley rat | Oddball paradigm | EEG – auditory cortex | Low 34/38 Hz evoked power to standards, baseline activity. | Chronic PCP (15 mg/kg /day for 4 weeks) or vehicle | Between-subject design. | Gamma-band power was reduced after PCP administration for standard stimuli. Baseline power was not affected. |
| Leishman et al., 2015 | N = 12 male Sprague-Dawley rats | 40 Hz ASSR paradigm (click trains) | EEG auditory cortex, cerebellum | 30, 40, 50 and 55 Hz ITPC and evoked power | Exp1: Acute: PCP (1.0, 2.5 and 4.0 mg/kg s.c.) or vehicle.  Exp2: acute (5 mg/kg), chronic continuous for 2 weeks (5 mg/kg/day). | Exp1: Pseudo-randomised, repeated-measure design.  Exp2: between-subject design | PCP increased 40 Hz ITPC at 1 mg/KG. Chronic PCP did not affect gamma-band activity. |
| Martin et al., 2017 | N = 31 (Exp1= 10, exp 2 = 21) male, adult Sprague-Dawley rats | Single tone 1 sec duration | EEG – temporal cortex | 39.3 -78.4 Hz ITPC and evoked power | PCP: continuous acute (1.0, 2.5, and 4.5 mg/kg s.c.) (Exp 1); chronic (5 mg/kg/day) for 14 days (Exp 2) or vehicle. | Exp1: Randomised order, repeated measure. Exp2: between-subject design, randomised. | Acute PCP decreased 50-70 Hz ITPC and spectral power (especially at higher doses). Chronic PC had no effect. |
| Raith et al., 2020 | N = 12 male adult Wistar rats | Oddball paradigm | EEG – auditory cortex | 20–50 Hz evoked power and ITPC. | Acute S-(+)-ketamine hydrochloride (i.p., 10 mg/kg), vehicle | Repeated measures not randomised. 3 days washout. | Reduction of evoked and ITPC gamma-band activity . |
| Raza et al., 2021 | N = 12 female Peri adolescent Sprague Dawley rats | A tone stimulus (50 ms ) followed 40 Hz ASSR ( click-train) | EEG – cortical recording | 30-100 Hz evoked power and ITPC . | Acute MK801 (0.025 and 0.05 mg/kg i.p.) or vehicle. | Repeated measure, randomised order (3-days washout) | MK801 attenuated evoked and ITPC gamma-band power. |
| Schuelert et al. 2018 | N = 12 adult male B6 mice | Paired Clicks, 40 Hz ASSR (click trains) | EEG-cortical recordings | 35-80 Hz evoked power and ITPC, baseline activity | Acute Ketamine (10mg/kg), MK-801, (0.04, 01, 0.3 per kg) | Repeated measures, randomised. | Ketamine had no effect on evoked gamma-band power, while only the largest dose of MK-801 (0.3 mg) impaired evoked gamma-band activity. Both Ketamine and MK-801 (0.1 and 0.3) reduced 40 Hz spectral power and ITPC. Baseline power was increased at higher dosages of MK-801 as well as Ketamine |
| Sivarao et al., 2016 | N = 12 male, adult Sprague-Dawley rats | 40 Hz ASSR (click train) | EEG (frontal, midline) | 35–45 Hz ITPC and evoked power . | Acute racemic ketamine hydrochloride (1, 3, 10, and 30 mg/kg; i.v.) or vehicle | Repeated measures, randomised. | ITPC: Increased at 10 mg/kg dose. The 30 mg/kg dose showed an initial decrease followed by a reversal at 62 mins after injection.  Evoked gamma-band power: increased at 10 mg/kg dose only.  Induced power: increased at 1 and 30 mg/kg |
| Sullivan et al., 2015 | N = 18 male, adult Sprague–Dawley rats | 40 Hz ASSR (click trains) | EEG –primary auditory cortex, HP | 20, 40, 80 Hz evoked power and ITPC analyses. | Acute: (bolus: MK-801 0.1 mg/kg, i.p.) Chronic: bolus injection for 21 consecutive days | Counterbalanced cross-over design. | Acute MK-801 treatment increased ITPC, while no effect was observed spectral power. |
