# Supplementary material for: Effects of N-Methyl-d-Aspartate Receptor Antagonists on Gamma-Band Activity During Auditory Stimulation Compared With Electro/Magneto-encephalographic Data in Schizophrenia and Early-Stage Psychosis: A Systematic Review and Perspective
Source: Schizophr Bull. 2024 Jun 27;50(5):1104–16. doi: 10.1093/schbul/sbae090 (PMC11349021; doi:10.1093/schbul/sbae090)
Supplement: sbae090_suppl_Supplementary_Material [file sbae090_suppl_supplementary_material.zip › SI Table 5_Human_Uhl_6.6.docx]

| **Table 5. *Summary of Studies in Human Participants*** | | | | | | | | |
| --- | --- | --- | --- | --- | --- | --- | --- | --- |
| Reference | Participants | Task type | Imaging technique | Analysis | Pharmacology | Study design | Symptom correlation | Main results |
| Curic et al., 2019 | N = 25 male participants | Auditory reaction task | 64 channel EEG | Source and sensor analysis.  Gamma (20-80 Hz). ITPC and evoked power | S- ketamine hydrochloride. Initial bolus (10 mg/ 5 min), continuous (0.006 mg/kg/min). | Placebo-controlled randomized cross-over study design. Single-blind. 7 days washout | Positive correlation between the PANSS negative score and the relative decreased gamma-band power following ketamine | Reduced auditory-evoked gamma-band power, ITPC and source gamma-band activity following ketamine compared to placebo. |
| Haaf et al., 2022 | N = 24 healthy male | Auditory reaction task | 64 channel EEG | Source and sensor 32-48 Hz ITPC and  evoked activity | Initial bolus S- ketamine hydrochloride (10mg/5 mins), continuous (.006 mg/kg/min, reduced by 10% every 10 minutes ), or vehicle | Double-blind, randomised, placebo-controlled crossover design. | Negative correlation between PANSS negative scores and auditory evoked gamma after ketamine. | Reduced evoked gamma-band activity ketamine following activity, especially in anterior cingulate cortex. |
| Hong et al., 2010 | N = 10 healthy participants (n = 4 females) | Paired-click paradigm | 6-channel EEG | Sensor 30-100 Hz evoked power . | Single bolus of Ketamine (0.3mg/kg. 1-minute infusion) or saline. 1 week washout. | Double-blind, placebo-controlled, crossover study | Withdrawal symptoms positively correlated with gamma-band activity (40-85 Hz) | Ketamine significantly increased gamma-band power compared to vehicle. |
| *Note.* ASSR, Auditory Steady State Response; EEG, Electroencephalography; MEG, Magnetoencephalography; MMN; Mismatch Negativity; ITPC, Inter-trial Phase Coherence; PCP, Phencyclidine; PPI; pre pulse inhibition., i.v., intravenous; ICA; independent component analysis; PANSS, Positive and Negative Syndrome Scale. | | | | | | | | |
