# Supplementary material for: Effects of N-Methyl-d-Aspartate Receptor Antagonists on Gamma-Band Activity During Auditory Stimulation Compared With Electro/Magneto-encephalographic Data in Schizophrenia and Early-Stage Psychosis: A Systematic Review and Perspective
Source: Schizophr Bull. 2024 Jun 27;50(5):1104–16. doi: 10.1093/schbul/sbae090 (PMC11349021; doi:10.1093/schbul/sbae090)
Supplement: sbae090_suppl_Supplementary_Material [file sbae090_suppl_supplementary_material.zip › SI Table SchZ Psychosis 6_6.6_Uhl.docx]

SI Table 6. Summary Table ScZ and Early-Stage Psychosis

| REFERENCE | Participants | Illness duration | Medication | Task | EEG/MEG | Analysis | Symptom correlation * | MAIN RESULTS |
| --- | --- | --- | --- | --- | --- | --- | --- | --- |
| Basar-Eroglu et al., 2011 | 10 chronic ScZ (mean age: 31.5) 10 HCs | 6.7 years | Medicated | Oddball paradigm | 8-channel EEG | Sensor, 25-45 Hz, evoked power | - | No difference in evoked gamma-band power. |
| Blumenfeld & Clementz, 2001 | 20 ScZ ( mean age: 36.6); 20 HCs | - | Mixed | Paired click paradigm | 148-channel MEG | Sensor and source 30-50 Hz evoked power | - | No significant difference between groups for evoked gamma-band power. |
| Brenner et al., 2003 | 21 ScZ or ScA ( mean age:45.6; 22 HCs | - | Mixed | 31, 42, 51, 62 71 and 82 Hz ASSR | 16-EEG | Sensor 31-82 Hz spectral (evoked) power  Artefact rejection: filtering |  | Reduced gamma-band power to 42, 51 and 62 Hz ASSR in ScZ-patients. |
| Brockhaus-Dumke et al., 2008 | 32 ScZ ( mean age: 31.9; 32 HCs | 3.3 | Mixed | Paired-click paradigm | 32-channel EEG | Sensor 33-60 Hz ITCP and evoked power . |  | ITCP and evoked gamma-band power were intact in ScZ-patients. |
| Edgar et al., 2013 | 39 ScZ ( mean age: 40.8) ; 29 HCs | Mixed | 40 Hz ASSR |  | 36-channel MEG | Source 38-42 Hz power and ITPC, total power (baseline, stimulation period) |  | Decreased ITPC in ScZ in the left STG but increased baseline total power in left STG and elevated 40 Hz power in right STG |
| Fujimoto, et al., 2013 | 10 ScZ-patients  (mean age: 30.9) 10 HCs | 7.1 | medicated | Oddball | 160-channel MEG, 19-channel EEG | Source 30-50 + 50-100 Hz induced power | Hallucinations and delusions  were positively correlated with lower gamma-band power while high gamma-band power correlated with conceptual disorganization | 30-50 Hz induced power was decreased in temporal, parietal, occipital and midline areas in ScZ. 50-100 Hz induced power was decreased in the eft occipital lobe. |
| Gallinat et al., 2004 | 15 ScZ-patients (mean age: 28.4); 15 HCs | 4.6 | Unmedicated | Oddball paradigm | 32-channel EEG | Sensor evoked and induced 40 Hz 33.64 Hz to 46.36 Hz power to standard and target stimuli | No correlations between late gamma response and PANSS | ScZ patients showed reduced evoked gamma-band power in a late-latency range to target stimuli over right frontal scalp regions. Induced gamma-band activity was intact. |
| Grent-‘t-Jong, et al., 2021 | 116 CHR-Ps (mean age: 22);  33 FEPs (mean age: 24); 49 HCs |  | Mixed | 40 Hz ASSR (amplitude modulated tones) | 248-channel MEG | Source 38-42 Hz ITCP and evoked power, naseline power |  | 40 Hz power and ITPC was reduced in CHR-P and FEP-groups. No differences in baseline activity. |
| Griskova-Bulanova et al., 2016 | 26 ScZ-patients (with and without AH) (mean age: 39 .5 ) 25 HCs |  | Medicated | 40 Hz ASSR (click trains) | 74-channel EEG | Sensor, 30-50 Hz ITPC and evoked spectral power | 40 Hz ITCP was positively correlated with positive symptoms and negative symptoms. Early-latency, power was negatively correlated with negative symptoms. | Early latency ITPC and evoked power were diminished in ScZ-patients |
| Hall et al., 2011 | 39 ScZ patients (mean age: 41.2) |  | Medicated | Oddball | 16-channel EEG | Sensor 35-46 Hz evoked power and ITPC | No correlations between SAPS and SANS with ITPCS and evoked power | Both ITPC and gamma-band power were reduced in schizophrenia patients. |
| Hamm et al., 2011 | 17 ScZ ( mean age: 40.7); 18 HCs | 18.2 | Mixed | 40, 80, 160 Hz ASSR | MEG: 128-channel | Sensor and source ITCP and evoked power, baseline activity | Correlation between negative sympoms and 80-Hz aSSR amplitude | Reduced ITPC and evoked power to 40 and 80 Hz ASSR, while deficits to 40 Hz ASSRs in evoked power were only detected in right auditory cortex. No differences in baseline activity. |
| Hamm et al, 2012 | 17 ScZ ( mean age: 41.5); 16 HCs |  | Mixed | 40 Hz ASSR | 256-channel EEG | Sensor; 30 -44 36-56 Hz ITCP and evoked and induced power . |  | Increased ITPC, evoked and induced power to 40 Hz ASSRs in ScZ-patients. |
| Hamm et al., 2015 | 18 ScZ (mean age: 45.6); 18 HCs |  | Mixed | 40 Hz ASSR (click trains) | 256-channel EEG | Sensor; 30-50 Hz evoked power . |  | ScZ patients displayed reduced 40-Hz ASSR evoked power. |
| Hayrynen et al., 2016 | 15 ScZ ( mean age = 44.8); 16 HCs |  | Mixed | Oddball-paradigm task during 40/88 Hz ASSRs | 248-channel MEG | Sensor 40 and 88 Hz evoked power. |  | ScZ patients showed greater 40 Hz evoked power in the left hemisphere during 40 Hz ASSRs without oddball targets. |
| Hirano et al, 2015 | 24 ScZ ( mean age: 46) 24 HCs ( | 21.1 | Medicated | 30, 40 Hz ASSR | 71-channel EEG | Source 30-33Hz /40-44 Hz evoked power and ITPC, 30-100 Hz induced power | ﻿40-Hz ITPC was not correlated with AH. Induced power positively correlated with AH in left hemispheres. | Reduced 40 Hz ASSR ITPC but intact evoked power. Increased mean induced gamma power in ScZ vs. HCs.  No differences in 30 Hz. |
| Hong et al., 2004 | 24 ScZ ( mean age: 39.7); 17 HCs |  | Medicated | 30 and 40 Hz ASSR (click trains) | 28-channel EEG | Sensor 25-45 Hz evoked power . | No correlations between 40 Hz synchronization power with BPRS and subscales | No difference in 30/40 Hz ASSR evoked power in ScZ-patients. |
| Khadimillah et tal., 2019 | 33 Early-Stage Psychosis Patients (mean age: 24.6), 33 Controls |  | Medicated | 40 Hz ASSR | 64-channel EEG | Sensor 38 -42 Hz evoked power and ITPC |  | ﻿Reduced ITPC and evoked power to 40 Hz ASSR,  particularly during the late-latency. |
| Kim et al., 2019 | 33 SZ (mean age: 42.21); 31 HCs |  | Medicated | 40 Hz ASSR | 64-channel EEG | Sensor and source 36-45 Hz ITPC, evoked, baseline and total power. |  | Increased ITPC, evoked and total power in ScZ patients.  No differences in baseline power. |
| Kirihara et al, 2012 | 234 ScZ (mean age: 44.5); 188 HCs | 22.7 | Mixed | 40 Hz ASSR | 34-channel EEG | Sensor 38-42 Hz ITPC and evoked power. | No correlations with cognitive and clinical measures | Reduced ITCP but intact evoked power in ScZ-patients. |
| Koshiyama, et al., 2020 | 427 ScZ (mean age: 45.5); 293 HCs | 23.6 | Mixed | 40 Hz ASSR (click trains) | 40 -channel EEG | Sensor and Source,  35-45 Hz ITPC | Negative correlations between ITPC and SAPS and SANS | Reduced 40 Hz ITPC in the left temporal and frontal cortex. |
| Krishnan et al, 2009 | 21 ScZ (mean age: 42.6); 21 HCs |  | Medicated | 30, 35, 40, 45 and 50 Hz ASSR | 29-channel EEG | Sensor ITPC and evoked spectral power. |  | ScZ-patients showed reduced gamma- band ITPC and spectral power across gamma-band frequencies. |
| Kwon et al., 1999 | 15 ScZ (mean age: 43.3); 15 HCs | 21.1 | Mixed | 30/40 Hz ASSR (click trains) | 64-channels EEG | Sensor; 30 and 40 Hz power and phase analysis. |  | Reduced evoked power at 40 Hz and phase delay in ScZ-patients. No differences for 30 Hz. |
| Leicht et al., 2010 | 90 ScZ (mean age:40.2), 90 HCs | 26.2 | Mixed | Auditory reaction paradigm | 27-channel EEG | Sensor and source, 32-48 Hz evoked power and ITPC | No correlation between power and ITPC with clinical symptoms | Reduced evoked gamma-band power (32-48 Hz) and ITPC in ScZ-patients. |
| Leicht et al., 2016 | 24 CHRs (mean age: 21.5); 24 HCs |  | Mixed | Auditory reaction task | 62-channel EEG | Sensor 30-50 Hz ITPC and evoked spectral power | No correlations. between : gamma-band responses and SOPS. | Gamma-band power was significantly reduced in CHR-Ps while deficits in ITPC only reached trend level. |
| Leicht et al., 2015 | 23 FEPs (mean age: 23.5) and 22 HCs | <1 | Mixed | Auditory reaction task | 66-channel EEG | Sensor; 32-48 Hz power and ITPC | Correlations between ITPC and 32-48 Hz power and negative symptoms as well as evoked power and disorganised factor. | Reduced evoked gamma-band power and ITPC in FEPs. |
| Light et al., 2006 | 100 ScZ-patients ( mean age: 42.5) 80 HCs | 17.95 | Mixed | 30/40 Hz ASSR | 34-channel EEG | Sensor; 26-45 Hz evoked power and ITPC | No correlations: SAPS and SANS with EEG measures | Reductions in 30- and 40-Hz evoked power and ITPC in ScZ-patients. |
| Nguyen et al., 2020 | 118 ScZ & 13 SA(mean age: 40)  196 healthy controls |  | Mixed | Paired lick- paradigm | 29-channel EEG | Sensor, 30-50 Hz evoked and induced power | No correlations between PANSS and EEG variables | ScZ-patients exhibited reduced evoked but intact induced gamma-band power. |
| Oribe et al., 2019 | 18 CHR-Ps (mean age: 21.2), 18 FEP-ScZ (mean age: 23.1) 40 HCs |  | Mixed | Oddball task | 68-channel EEG | Sensor, 40-100 Hz ITPC and evoked to standard trials |  | No differences between groups, only at follow-up in FEP-ScZ. |
| Parker et al., 2019 | 113 ScZ, SA, BPD (mean age: 39); 137 HCs |  | Mixed | 40/80 Hz ASSR | 64-channel EEG | Sensor 40 and 80 Hz ITPC and evoked. Baseline power analysis | No correlations between ITPC/power/ and symptom ratings | ScZ-patients showed reduced ITPC and evoked power towards 40 Hz and 80 Hz ASSRS as well as increased gamma-band baseline power. |
| Perez et al., 2013 | 19 ScZ-patients ( mean age: 23.9) ; 43 CHR-P ( mean age: 16.8 ); 42 HCs |  | Mixed | Oddball paradigm | 20-channel EEG | Sensor; 3 0-60 Hz evoked power and ITPC | No correlations: PANSS and SOPS with EEG measures | Gamma-band ITPC was significantly reduced in both ScZ and CHR-Ps, while evoked power was also decreased in ScZ-patients, reaching only a trend-level in CHR-Ps |
| Popov et al., 2011 | 50 ScZ ( mean age: 30.2); 48 HCs ( mean age: 28.1) |  | M edicated | Paired click paradigm | MEG: 148-channel | Sensor and source data, 60-80 Hz e evoked and induced power to S1. |  | Reduced 60–80 Hz evoked power in ScZ-patients  ﻿in left superior frontal gyrus, left middle frontal gyrus, and left precentral sulcus  No differences in induced power. |
| Puvvada et al., 2017 | 128 ScZ-patients ( mean age: 37.8); 108 HCs |  | Mixed | 40 and 80 Hz ASSR | 64 -channel EEG | Sensor, 40 and 80 Hz ITPC and evoked power | No correlations between  BPRS-ratings with ASSRs at any frequency | Reduced 40 Hz  evoked power in ScZ-patients but no difference at 80 Hz. For ITPC 40 and 80 Hz were reduced. |
| Rass et al., 2012 | 42 ScZ & ScA ( mean age: 36.86); 56 HCs |  | Mixed | 30, 40 and 50 Hz ASSR (click trains) | 32-channel EEG | Sensor 30,40,50 Hz mean trial power (evoked) and ITPC |  | ScZ-patients showed a reduction in evoked power to 40 Hz ASSR at trend level. |
| Roach et al., 2008 | 21 ScZ ( mean age: 39.2), 22 HCs |  | Medicated | Oddball task | 26-channel EEG | Sensor 36-50 Hz ITPC ; | No correlations with  clinical symptoms with 40-Hz ITPC | Gamma-band ITPC over frontal electrodes was reduced in ScZ-patients |
| Spencer et al., 2008a | 16 FEP-ScZ (mean age: 25.5); 33 HCs |  | Medicated | 30 and 40 Hz ASSR (click trains) | 60-channel EEG | Sensor; 31.3-34.1, 40.5-44.1 Hz evoked power and ITPC | Phase locking of the 40 Hz harmonic of the 20 Hz ASSR was correlated with the Positive Symptom Total | Reduced 30 and 40-Hz ITPC and evoked power in ScZ-patients. |
| Spencer et al., 2008b | 23 ScZ ( mean age: 40.7), 21 HCs | 22.6 | Medicated | Oddball paradigm | 60-channel EEG | Sensor 0-100 Hz evoked power and ITPC |  | ITPC and evoked gamma-band power were not reduced in ScZ-patients. |
| Spencer et al, 2009 | 18 ScZ ( mean age: 39.8); 16 HCs |  | Medicated | 40 Hz ASSR (click trains) | 60-channel EEG | Sensor and source 38-50 Hz evoked power and ITPC | ITPC and evoked power were positively correlated with auditory hallucinations (SAPS) | ITPC and evoked power were reduced in ScZ-patients in left and right auditory cortex. |
| Tada et al., 2016 | 15 CHR-P (mean age: 21.1), 13 FEP (mean age: 22.1); 21 HCs |  | Medicated | 30 and 40 Hz ASSR (click trains) | 64-channel EEG | Sensor 26-45 Hz ITPC and evoked power | The late-latency 40 Hz ITPC and induced power correlated with general PANSS scores.  The early-latency 40 Hz ERSP correlated with positive symptoms and general PANSS ratings. | CHR-Ps had reduced late-latency ITPC, while FEPs had reduced both early- and late-latency ITPC to 40 Hz ASSRs while 30 Hz ASSRs were intact. |
| Taylor et al., 2013 | 28 FEP-patients (mean age: 24.8); 44 HCs |  | Medicated | Odball-paradigm | 60-channel EEG | Sensor 35-45 Hz ITPC and evoked power. | Evoked power correlated with positive symptoms,  ITPCS with total PANSS. | ITPC and evoked gamma-band power (35-45 Hz) were reduced in ScZ-patients. |
| Teale et al., 2008 | 15 ScZ (mean age: 37.9); 15 HCs | 12.6 | Medicated | 40 Hz ASSR | 248-channel MEG | Source; 35-45 Hz ITPC , e voked and induced power | No correlation: BRPS and any 40 Hz measures | Reduced ITPC and evoked power in auditory cortex at 40 Hz in ScZ-patients, while induced power was increased. |
| Tsuchimoto et al, 2011 | 17 ScZ ( mean age: 35.6); 22 HCs | 13.5 | M edicated | 30, 40 and 80 Hz ASSR (click trains) | MEG: 306-channel | Sensor 25-85 evoked power and ITPC | Negative correlation between left 80 Hz-ASSR-power and  auditory hallucination scores | ScZ-patients showed reduced power at 40-Hz while ITPC was intact. At 80-Hz both ITPC and mean evoked power were decreased. No differences in 30 Hz ASSR. |
| Vierling- Claassen et al, 2008 | 12 ScZ-Patients, 12 HCs | 26.2 | Medicated | 30 and 40 Hz ASSR (click trains) | 306-channel MEG | Source 30 and 40 Hz evoked power |  | Decreased 40 Hz but intact 30 Hz ASSRs evoked power in ScZ patients. |
| Wang et al., 2018 | 33 FEP-ScZ (24.7), 28 HCs | 4.6 months | Mixed | 40 Hz ASSR (click trains) | 64-channel EEG | Sensor, 36-45 Hz ITPC and induced power. | No correlations with PANSS scores and ITPC/power | FEPs showed lower ITPC and induced gamma-band power. |
| Wilson et al, 2008 | ﻿Early-Onset Psychosis (mean age: 14.64), 10  HCs |  | Mixed | 40 Hz ASSR  (click trains) | 248-channel MEG | Source 40 Hz evoked and baseline power |  | Decreased 40 Hz power but no differences in baseline activity. |
| Zhou et al., 2018 | ﻿52 ScZ-patients 55 ScA-patients  75 HC |  | Mixed | 30 and 40 Hz ASSR (click trains) | 18-channel EEG | Sensor 28 – 47 Hz, Hz ITPC and evoked power. | Evoked power and ITPC at 30 and 40 Hz correlated negatively with more severe hallucinations | ScZ and ScA-patients were characterized by lower ITPC and evoked power to 30 and 40 Hz ASSRs. |

*Note.* ASSR, auditory steady-state response; AVH, auditory verbal hallucinations; CAARMS, Comprehensive Assessment of At-Risk Mental States; CHR-P, clinical high-risk for psychosis; EEG, Electroencephalography; FEP, First-episode psychosis; HC, healthy controls; ICA, independent component analysis; ITPC, inter-trial phase coherence; MEG, Magnetoencephalography; MMN, mismatch negativity; NH, never hallucinating patients; PANSS, Positive and Negative Syndrome Scale; PLF, phase-locking factor; SAPS, Scale for the Assessment of Positive Symptoms; SANS Scale for the Assessment of Negative Symptoms; ScZ. Schizophrenia, ScA: Schizoaffective Disorders, SOPS, Scale of Prodromal Symptoms.
