# Supplementary figures and images for: Effects of N-Methyl-d-Aspartate Receptor Antagonists on Gamma-Band Activity During Auditory Stimulation Compared With Electro/Magneto-encephalographic Data in Schizophrenia and Early-Stage Psychosis: A Systematic Review and Perspective
Source: Schizophr Bull. 2024 Jun 27;50(5):1104–16. doi: 10.1093/schbul/sbae090 (PMC11349021; doi:10.1093/schbul/sbae090)

SI Figure 7. Effect Size Analysis for Evoked Gamma-Band Power in ScZ and Early-Stage Psychosis


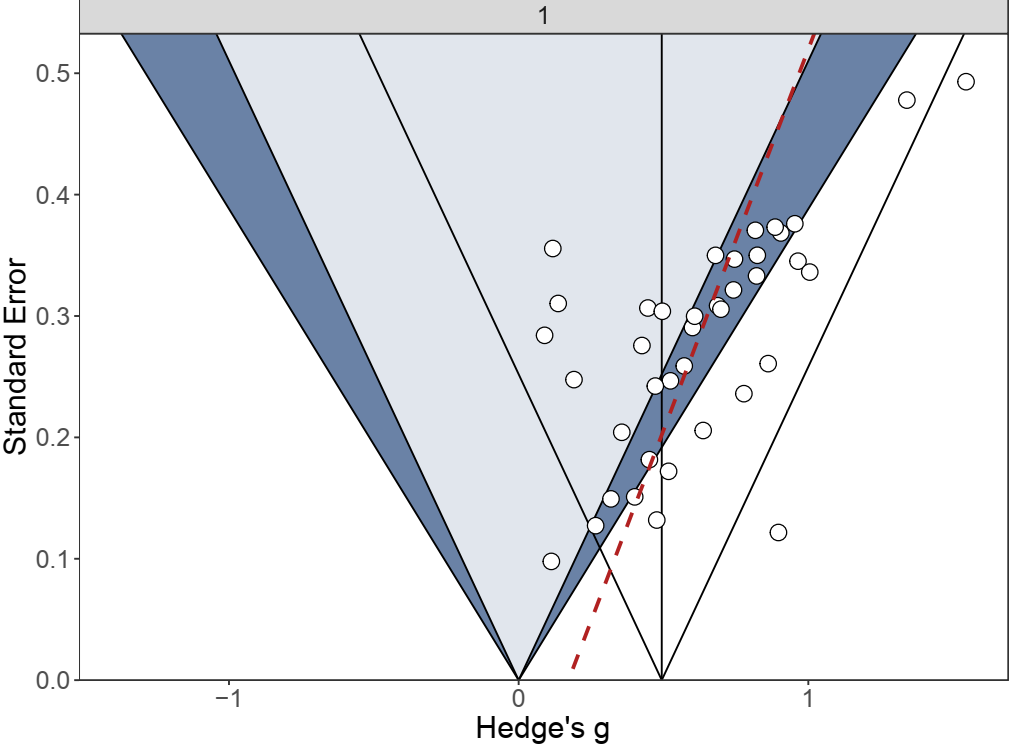


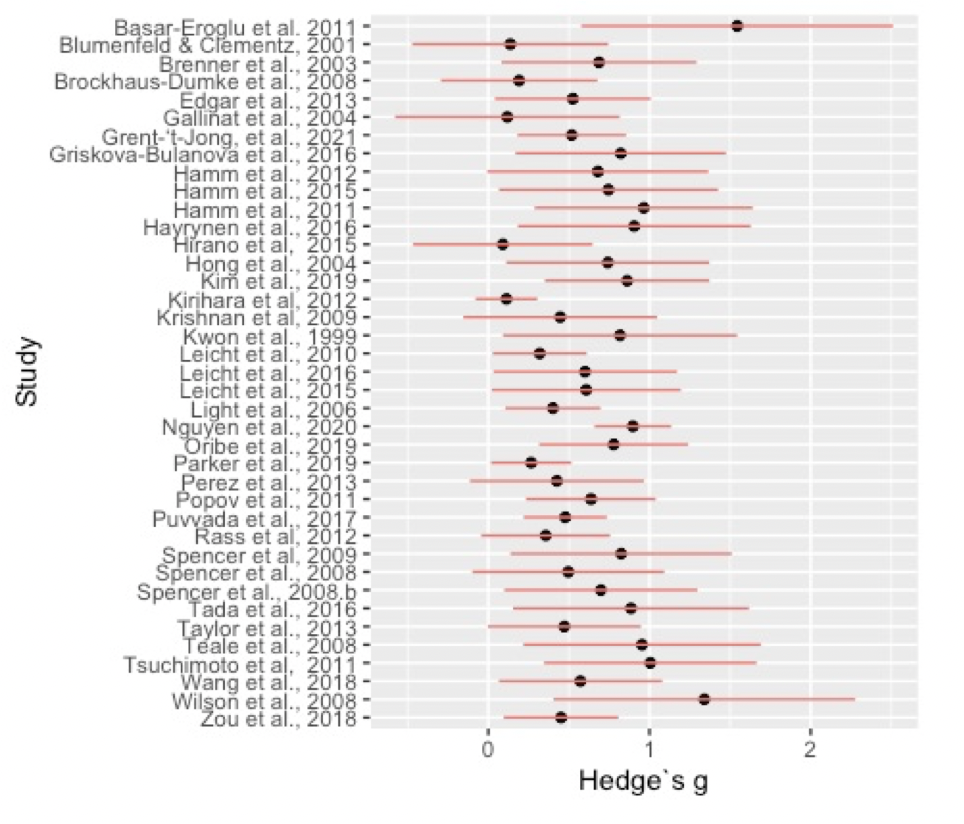

Supplement: sbae090_suppl_Supplementary_Material [file sbae090_suppl_supplementary_material.zip › SI Figure 3.docx]
